# Peptide-Hitchhiking for the Development of Nanosystems in Glioblastoma

**DOI:** 10.1021/acsnano.4c01790

**Published:** 2024-06-11

**Authors:** Francisco Branco, Joana Cunha, Maria Mendes, Carla Vitorino, João J. Sousa

**Affiliations:** † Faculty of Pharmacy, University of Coimbra, Pólo das Ciências da Saúde, Azinhaga de Santa Comba, 3000-548 Coimbra, Portugal; ‡ Coimbra Chemistry Centre, Institute of Molecular Sciences − IMS, Faculty of Sciences and Technology, University of Coimbra, 3004-535 Coimbra, Portugal

**Keywords:** glioblastoma, blood−brain barrier (BBB), peptide functionalization, nanoparticles, external stimuli, biomimetic approaches, extracellular vesicles, nanocatalytic medicine, nose-to-brain delivery, local treatment

## Abstract

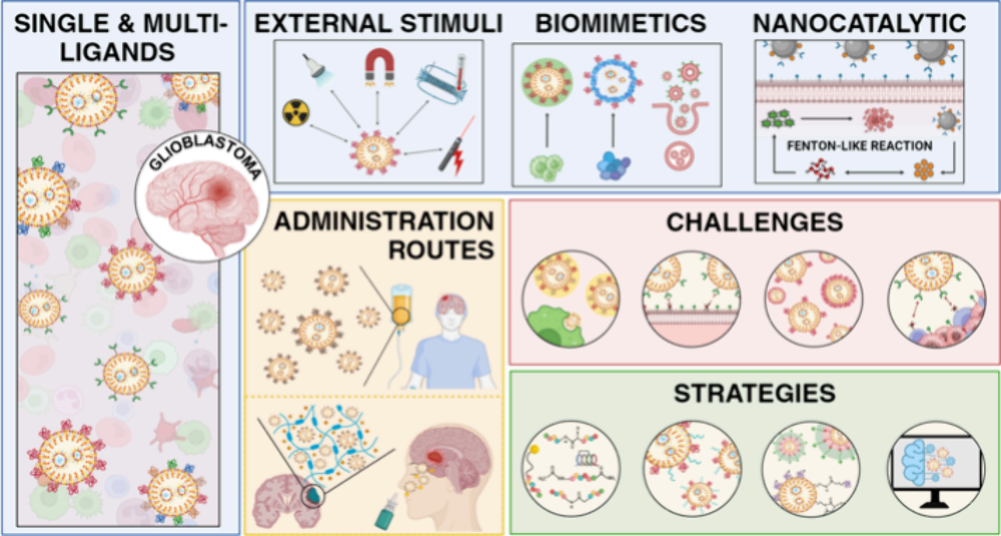

Glioblastoma (GBM) remains the epitome of aggressiveness and lethality in the spectrum of brain tumors, primarily due to the blood–brain barrier (BBB) that hinders effective treatment delivery, tumor heterogeneity, and the presence of treatment-resistant stem cells that contribute to tumor recurrence. Nanoparticles (NPs) have been used to overcome these obstacles by attaching targeting ligands to enhance therapeutic efficacy. Among these ligands, peptides stand out due to their ease of synthesis and high selectivity. This article aims to review single and multiligand strategies critically. In addition, it highlights other strategies that integrate the effects of external stimuli, biomimetic approaches, and chemical approaches as nanocatalytic medicine, revealing their significant potential in treating GBM with peptide-functionalized NPs. Alternative routes of parenteral administration, specifically nose-to-brain delivery and local treatment within the resected tumor cavity, are also discussed. Finally, an overview of the significant obstacles and potential strategies to overcome them are discussed to provide a perspective on this promising field of GBM therapy.

## 1 Introduction

Glioblastoma (GBM), classified as a grade IV glioma by the World Health Organization, is the most prevalent malignant tumor of the central nervous system (CNS), representing approximately 50.1% of all cases.^1,2^ Despite the advances in therapeutic approaches over the years, GBM remains the epitome of aggressiveness and lethality in the spectrum of brain tumors, with an expected 5-year survival rate of 6.9% and a median survival of 14 months from diagnosis.^2,3^ The current treatments for GBM include tumor resection, followed by radiation therapy (RT) and chemotherapy with Temozolomide (TMZ).^4,5^ However, the efficacy of these treatments is limited by several factors: (i) the infiltrative nature of GBM makes complete surgical resection nearly impossible, leaving residual tumor cells that contribute to recurrence;^6^ (ii) the lack of treatment specificity. Note that while RT effectively controls tumor growth, it can also damage surrounding healthy brain tissue/cells, resulting in significant side effects and promoting the suspension of treatment;^7^ (iii) the multidrug resistance, since the efficacy of chemotherapy is limited by the development of drug resistance mainly due to the presence of glioma stem cells (GSCs).^8^ GSCs play a pivotal role in the poor prognosis of GBM, keeping stem-like characteristics that contribute to tumor recurrence and resistance. This is attributed to their ability for self-renewal, treatment resistance, and adept capacity to infiltrate and migrate within the surrounding brain tumor.^9,10^ Finally, (iv) the presence of the blood–brain barrier (BBB), which limits the efficacy of chemotherapeutic drugs (e.g., immunotherapy approaches). The BBB acts as a gatekeeper to prevent harmful substances from entering the brain and presents a significant challenge to GBM treatment. Indeed, only 2% of small-molecule therapeutics and macromolecular drugs can cross BBB and access the brain, raising substantial challenges in neuro-oncology.^11,12^ The BBB primarily comprises endothelial cells (ECs), distinct in their lack of fenestrations compared to other barriers and tightly interconnected by junctional complexes (adherent junctions and tight junctions). These ECs are surrounded by pericytes and astrocytes, which are responsible for the homeostasis of the extracellular space and the maintenance of BBB integrity, respectively.^13^ Nevertheless, the tumoral environment is characterized by abnormal pericyte distribution and loss of astrocytic endfeet and neuronal connections. This leads to forming a vasculature known as the blood-tumor barrier (BTB).^14^ Although the BTB is more permeable than the BBB, it still presents significant hurdles for drugs to reach brain tumor sites by passive transport, relying on solid tumors’ enhanced permeability retention (EPR) effect. However, the effect of EPR depends highly on tumor type and location.^15−17^ All these factors dictate the urgent need for developing more efficient therapeutic drug delivery systems.

Nanomedicines have shown promise in overcoming these challenges by exploiting their unique properties. They may enhance the stability and solubility of the payload, endow the drugs with the ability to cross the BBB/BTB, and mitigate side effects.^18−20^ This internalization is achieved through diverse transcytosis pathways, with receptor-mediated transcytosis (RMT) emerging as the most promising and widely used method for delivering therapeutics to the brain.^21,22^ Therefore, nanoparticles (NPs) can be used as complex delivery systems to surpass biological obstacles and address patient heterogeneity. This is achieved by incorporating targeting ligands designed to target specific cellular and molecular pathways, consequently enhancing therapeutic efficacy.^23−25^ Among these ligands, peptides stand out as a popular and practical choice once they can be easily obtained and exhibit high selectivity and affinity toward their targets.^26^

This review focuses on the latest and most innovative drug delivery strategies based on the peptide functionalization of NPs for GBM treatments. It meticulously explores the diversity of approaches described in the literature, providing a detailed description of the most used peptides for each receptor, the cell-penetrating peptides (CPPs) employed as single ligands, and an analysis of peptide combinations. Additionally, we delve into peptide-modified biomimetic approaches, offering an updated perspective on mimicking biological processes for more effective GBM treatments. We particularly emphasize the significant promise and impact of combining external stimuli with peptide surface modification to enhance the delivery and efficacy of therapeutic agents for GBM. We also examine the synergy between nanocatalytic medicine, an emerging field that leverages the catalytic capabilities of NPs for therapeutic applications, and peptide surface modification, revealing a promising approach. Finally, we discuss the main challenges in formulating these peptide-functionalized NPs and present some strategies to overcome them.

## 2 Beyond the Surface: Peptide Functionalization Strategies for Enhanced GBM Therapy

### 2.1 One Ligand, Multiple Horizons: Focusing on Single Ligand Pathways

**Figure 1:**
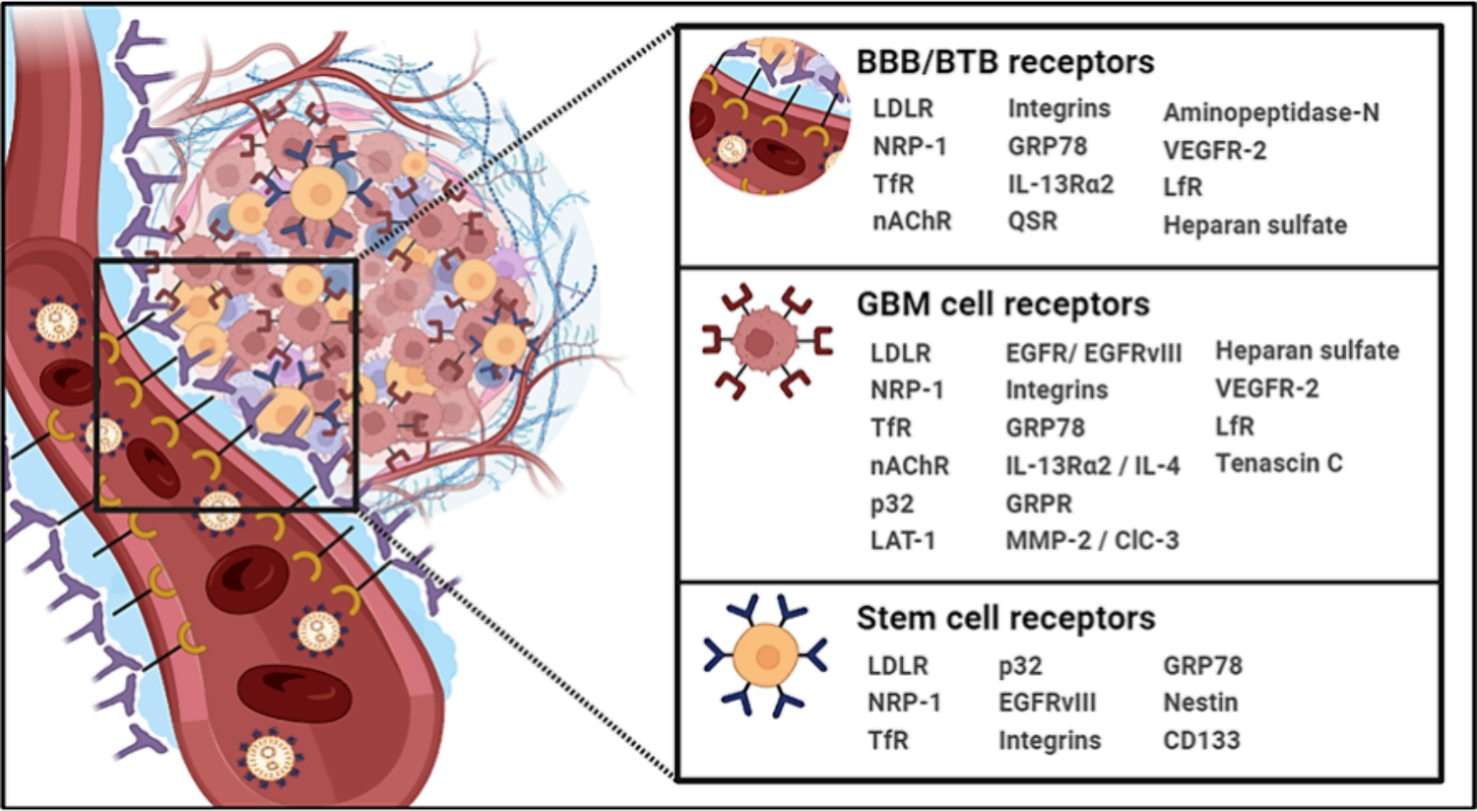
Receptor profiling in glioblastoma. Schematic representation of critical receptors overexpressed in the BBB, GBM cells, and stem cells that have been exploited as targets for precision GBM therapy. Abbreviations: BBB: Blood-brain barrier; BTB: Blood tumor barrier; GBM: Glioblastoma. (Created with BioRender.com).

In targeted therapy, a unique receptor on specific cell types enables the precise direction of nanosystems using tailored ligands. Moreover, within tumor microenvironments, the overexpression of receptors across a range of cells allows for a single ligand to concurrently target multiple cell variants, thereby enhancing the efficiency of the targeting process.^27^ The upcoming sections will address receptors exploited as targets in GBM treatment. (Figure 1) The discussion will detail how NP systems with a single ligand peptide can effectively target multiple elements, akin to the “two birds with one stone” strategy in most scenarios. Moreover, the analysis will cover specific strategies in which peptides target the BBB and GBM cells and affect the ability to target GSCs.

#### 2.1.1 Low-Density Lipoprotein Receptor

The low-density lipoprotein receptors (LDLRs) family, responsible for cholesterol homeostasis by uptaking circulating cholesterol-containing lipoprotein particles, has been identified as overexpressed in brain ECs and GBM cells. However, neurons and normal brain tissues have relatively low expression of LDLRs, which makes these receptors significant biomarkers of GBM.^28,29^ Indeed, some studies have reported that despite their overexpression in the BBB and GBM cells, they are not overexpressed in astrocytes and microglia.^30,31^ Within the wide range of receptors belonging to the LDLR family, we highlight the importance of LDLR, LDLR-related protein-1 (LRP-1), and megalin (LRP-2) due to their primary influence in targeting and binding to peptide ligands, as will be shown in the following sections.^32^

Angiopep-2 (ANG2), a synthetic peptide derived from the Kunitz domain of the LRP-1 ligands, demonstrated a high BBB transcytosis efficacy by targeting LRP-1.^33,34^ Several studies have shown that the functionalization of NPs with ANG2 enhances BBB penetration and GBM cell targeting. For instance, Straehla *et al.* conjugated ANG2 to the surface of cisplatin-loaded liposomes through a layer-by-layer assembly, demonstrating improved efficacy in an *in vitro* microfluidic platform of vascularized GBM spheroids. The translatability of this model was verified in an *in vivo* orthotopic xenograft model of patient-derived GBM cells.^35^ Moreover, solid lipid nanoparticles (SLNs), polymeric NPs, and chimeric polymersomes functionalized with ANG2 have shown enhanced efficacy in GBM treatment in both *in vitro* and *in vivo* studies.^36−39^

Apolipoprotein E (ApoE) peptide presents another targeting opportunity for LDLR. ApoE-modified saporin-loaded chimeric polymersomes showed a highly efficient crossing of the BBB and accumulation in GBM. This was related to their multireceptor targeting properties to LRP-1, LRP-2, and LDLR. These polymersomes leveraged BBB transcytosis and were internalized into GBM cells through receptor-mediated endocytosis.^40^ ApoE diverse receptor targeting has been validated in other approaches, with *in vivo* results demonstrating survival benefits and low side effects.^41−43^

A chaperone protein named receptor-associated protein (RAP) binds to LRP-1. Exploiting this property for GBM therapy, Ruan and co-workers designed a miniaturized RAP protein using a Monte Carlo-based algorithm, resulting in the development of the RAP12 peptide (EAKIEKHNHYQK). RAP12 was used to modify paclitaxel (PTX) loaded-poly(ethylene glycol)-*block*-poly(lactic acid) (PEG–PLA) micelles, exhibiting a solid ability to cross the BBB and target GBM. These findings led to an inhibition of GBM cell growth and an extension of the median lifespan of nude mice, suggesting the potential effectiveness of RAP12 as a ligand for GBM treatment.^44^ Moreover, in other studies, the use of a derivative of RAP-12, the stapled RAP-12 (sRAP12) peptide, demonstrated successful penetration of the BBB/BTB and targeted tumor sites in both *in vitro* and *in vivo* studies. This resulted in an extended median survival time in the orthotopic glioma-bearing mice model.^45^

#### 2.1.2 Transferrin Receptor

The transferrin receptor (TfR) plays a critical role in binding and sequestering iron from the bloodstream, regulating its concentration in body fluids. Its overexpression in both brain ECs and GBM cells has made TfR an attractive target for GBM.^46^ Moreover, while endothelial cells of the BBB express these receptors, astrocytes and oligodendrocytes are reported not to express TfR directly.^47^

In recent studies, poly(lactide-*co*-glycolide) (PLGA) NPs modified with transferrin (Tf) loaded with a combination of TMZ and bortezomib, an O^6^-methylguanine-DNA methyltransferase (MGMT) protein downregulating drug, presented a strategy to overcome drug resistance problems and improve TMZ efficacy. Tf conjugation improved targeting through BBB and increased the specificity of cargo delivery for GBM cells.^48^ Interestingly, TfR has been found to be overexpressed in GSCs, which suggests a potential strategy for treating GBM. Sun and co-workers modified polymeric dendrimers with Tf, proving to be an efficient target for both GBM cells and GSCs, resulting in delayed tumor recurrence in nude mice models, possibly attributed to the elimination of GSCs.^49^

Nevertheless, a significant challenge in TfR-guided targeting is the competition between exogenous and endogenous Tf.^50^ Therefore, other strategies have been devised to overcome this issue. The T7 (His-Ala-Ile-Tyr-Pro-Arg-His) peptide was identified to have a similar targeting ability without competing with endogenous Tf for receptor binding. T7 peptide-functionalized carmustine-loaded PLGA NPs specifically targeted Tf receptors overexpressed in the BBB and GBM cells, concentrating the NPs mainly in the tumor with minimal toxicity and side effects. *In vivo* studies also showed the smallest tumor size and significantly longer survival in the targeted group.^51^ Also, functionalization of PTX-loaded PLGA NPs with CRT (CRTIGPSVC) peptide, an allosteric TfR binding peptide, has revealed enhanced accumulation in GBM cells and prolonged mouse survival compared to nonfunctionalized NPs.^52^ Additionally, PEG–PLA micelles modified with TfR-T12 (THRPPMWSPVWP) peptide were designed, with both *in vitro* and *in vivo* results revealing the efficacy of this peptide in crossing BBB and target tumor cells.^53^

#### 2.1.3 Neuropilin-1 Receptor

Neuropilin-1 (NRP-1) is a transmembrane protein highly expressed on the surface of GBM cells and the ECs forming part of angiogenic blood vessels.^54^

The tLyp-1 (CGNKRTR) peptide, known for its high-affinity binding peptide for NRP-1 through a C-end rule (CendR) internalization pathway, was considered a dual-targeting ligand for GBM. Hu *et al.* functionalized PEG–PLA NPs with tLyp-1, demonstrating enhanced cellular uptake in C6 glioma cells and improved payload efficacy, thereby validating the dual-targeting capabilities of this peptide.^55^ In two different approaches, the surface of niosomes was modified with tLyp-1, and both *in vitro* results showed increased cytotoxicity of the drugs.^56,57^ Wang and colleagues recently developed tLyp-1 nanoliposomes containing TMZ, which, when tested *in vivo* in nude mice, demonstrated the capability of tLyp-1 to traverse the BBB and effectively penetrate tumor cells through binding with the NRP-1 receptor.^58^

In the context of GBM, the mitochondrial protein p32 is overexpressed at the surface of both tumor and GSCs.^59,60^ The LinTT1 (AKRGARSTA) peptide, known for its tumor-homing properties and high affinity to the p32 protein, was exploited by Säälik and co-workers in an innovative approach. Iron oxide nanoworms (NWs) were functionalized with LinTT1, and after targeting and being internalized by the p32 protein, they were proteolytically processed. The cleavage of the LinTT1 peptide exposed a CendR motif that binds NRP-1. This strategy is interesting as p32 is overexpressed in normal GBM cells and GSCs, suggesting increased NP uptake and improved therapeutic efficacy.^61,62^

In a recent study, the iNGR peptide was conjugated to PTX-loaded NPs within a poly(ethylene glycol)-*block*-poly(lactide-*co*-glycolide) (PEG–PLGA) matrix. After initially binding to the enzyme aminopeptidase N, specifically upregulated in angiogenic blood vessels and surrounding pericytes, the iNGR peptide was proteolytically cleaved into CRNGR. This cleaved peptide interacted with the NRP-1, facilitating deep penetration into the tumor tissue, enhancing antiangiogenic effects, and notably extending the survival of mice with intracranial glioma.^63^

#### 2.1.4 Interleukin-13 Receptor Alpha 2

Interleukin 13 receptor alpha 2 (IL-13Rα2) has been extensively investigated in GBM and is reported to be overexpressed in three out of four glioma patients.^64^ Additionally, the absence of IL13Rα2 in the normal tissues surrounding GBM enhances its suitability as a target. Several approaches using peptide ligands to functionalize NPs have been employed to target this receptor.^65^ Gao *et al.* modified the surface of docetaxel (DTX)-loaded lipid NPs with IL-13 peptide, proving its effectiveness as a ligand for IL-13Rα2 in treating GBM. Results showed an increased intracellular delivery of NPs, precise targeting of GBM cells, and a significant extension of the median lifetime of mice.^66^

The Pep-1 (CGEMGWVRC) peptide was also used as a binding ligand of IL-13Rα2 in targeting NPs, exhibiting high targeting efficiency and specificity for GBM cells.^67−69^ Jiang *et al.* designed PEGylated polyamidoamine dendrimers modified with Pep-1, where this peptide served as an anchor to enhance the delivery of NPs across BTB and target them specifically to glioma cells. Both *in vitro* and *in vivo* demonstrated that these functionalized dendrimers exhibited enhanced cellular uptake and effective penetration into tissues.^70^ The same group proposed a different approach and developed self-assembled disulfide bond PTX prodrug NPs conjugated with Pep-1 peptide. This delivery system demonstrated a high cargo loading capacity (56.03%) and proved stable under physiological conditions, preventing drug leakage and minimizing the toxicity associated with off-target effects. Moreover, they exhibited redox-responsive characteristics once the NPs penetrated the tumor microenvironment via IL-13Rα2-mediated internalization. These NPs were sensitive to the elevated glutathione levels found in GBM cells, triggering the release of PTX and significantly improving their antitumor efficacy.^71^

#### 2.1.5 Integrin Receptors

Integrins are transmembrane receptors composed of two subunits, α and β, capable of forming 24 different heterodimers.^72^ Among them, integrins α_v_β_3_ and α_v_β_5_ have been identified as key targets in GBM due to their roles in tumor growth and vascularization, with α_v_β_3_ being particularly prominent in high-grade gliomas and associated with poorer outcomes.^73,74^ Immunochemistry analyses have shown that integrins α_v_β_3_ are overexpressed in GBM cells, in contrast to their absence in normal brain tissue.^75^

Song *et al.* developed a TMZ-loaded nanostructured lipid nanocarrier decorated with Arginine-Glycine-Aspartic (RGD) peptide, a well-known binding ligand for integrins α_v_β_3_ and α_v_β_5_, enhancing the efficiency of drug delivery both *in vitro* and *in vivo*.^76^ Moreover, cyclic Arginine-Glycine-Aspartic acid (cRGD) peptide has been exploited to target the α_v_β_3_ and α_v_β_5_ integrins overexpressed on the ECs of GBM and has been applied as a targeting ligand to polymeric micelles and SLNs, improving the efficacy of GBM treatment in these approaches.^77−80^ Chauhan and co-workers designed pitavastatin-loaded SiO_2_ polymeric micelles decorated with cyclic Arginine-Glycine-Aspartic acid-(D)-phenylalanine-Valine (c(RGDfV)) peptide to target α_v_β_3_ in the BBB and in GBM cells, boosting the uptake of NPs and enhancing the antiproliferative effects of the cargo in pediatric patient-derived cells.^81^

In another study, PTX-loaded SLNs were functionalized with the internalizing RGD (iRGD, CRGDRGPDC), a tumor-homing peptide with a CendR motif. Initially, iRGD bound to the integrin α_v_β_3_, followed by the exposure of the CendR motif through tumor-derived protease cleavage, serving as a binding site for the NRP-1 receptor. Studies conducted with U87 cells in 2-dimensional (D) and 3D models demonstrated enhanced uptake by tumor cells and improved cytotoxic effects.^82^ Moreover, Ruan *et al.* designed a stapled RGD (sRGD) peptide to enhance the penetration of the BBB/BTB and improve targeting capabilities toward GBM. In this context, PTX-loaded PEG–PLA micelles successfully crossed the BBB/BTB and targeted GBM cells both *in vitro* and *in vivo*. Furthermore, this strategy significantly prolonged the median lifespan of orthotopic GBM-bearing mice.^83^

#### 2.1.6 Other Receptors

Although less commonly used, other receptors have also been shown to be viable targets for GBM. Nicotinic acetylcholine receptors (nAChRs) play a crucial role in the brain by modulating the transport and signaling of the neurotransmitter acetylcholine.^47^ While their overexpression in GBM compared to normal cells is not fully established, nAChRs have been used to target the BBB/BTB and enhance glioma treatment. These receptors are ligand-gated ion channels that, once bound, can reduce the transvascular delivery of drugs into the brain.^84^ Based on the understanding that nAChRs serve as a molecular target for rabies virus (RV), a rabies virus glycoprotein (RVG)-derived peptide was employed to mimic RVs transient pathway across the BBB for targeted delivery to GBM.^85,86^ Additionally, α7 nAChR, a member of this receptor family, is being explored as a potential target for brain tumors.^87^ Therefore, the ^D^CDX (GREIRTGRAERWSEKF) peptide was designed to bind to this receptor, and two different liposomal approaches were successfully functionalized, demonstrating that ^D^CDX facilitated BBB penetration and increased tumor accumulation, thus improving therapeutic efficacy.^88,89^

Based on data from The Cancer Genome Atlas GBM database, more than 50% of GBM patients exhibit either overexpression or mutations in epidermal growth factor receptor (EGFR).^90^ The most frequent mutation, EGFR variant III (EGFRvIII), is present in one-fourth of GBMs with EGFR amplification but is absent in normal tissues.^91^ Additionally, this receptor variant has been revealed to be upregulated in GSCs.^92^ Therefore, Mao and colleagues constructed PTX-loaded PEG–PLA micelles modified with D-AE, a selective ligand to both EGFR and EGFRvIII. This resulted in precisely targeting BTB, GBM cells, and GSCs, enhancing therapeutic effectiveness.^93^

Besides, Lv *et al.* used CGKRK peptide, a well-known binding ligand for heparan sulfate overexpressed in GBM cells, to modify the surface of the previously described self-assembled disulfide bond PTX prodrug NPs.^94^

Chlorotoxin (CTX), a scorpion venom-derived peptide consisting of 36 amino acids, exhibited a strong affinity for both matrix metalloproteinase 2 (MMP-2) and chloride channel-3 (ClC-3), which are significantly overexpressed in GBM cells but absent in healthy brain tissue.^95,96^ NPs conjugated with CTX showed promise as a strategy for targeted GBM delivery. Although the exact target of CTX remains unclear, MMP-2 appeared to be the main responsible for the uptake of NPs.^97−99^

The Glucose-Regulated Protein 78 (GRP78) is highly expressed in blood vessel endothelium, GBM cells, and GSCs but not abnormally expressed in normal cells, which positions cell-surface GRP78 as an ideal target for GBM targeting. Consequently, Ran *et al.* developed PTX-loaded PEG–PLA micelles modified with the three different VAP peptides, known for their binding solid affinity to GRP78. Both *in vitro* and *in vivo* studies demonstrated enhanced antitumor effects with these modified micelles.^100^

#### 2.1.7 Cell-Penetrating Peptides

Cell-penetrating peptides (CPPs) are short peptides (5–30 amino acids) recognized for their substantial ability to transduce cell membranes through a receptor-independent manner, making them valuable as drug delivery systems, particularly in directing chemotherapy treatments to GBM cells.^101^ CPPs have been classified into three subcategories according to their physicochemical properties: cationic, amphipathic, and hydrophobic.^102,103^ Most recognized CPPs are cationic and derived from insect, viral, or mammalian membrane translocating proteins.^104^ Various nonendocytic mechanisms have been reported to be involved in CPP internalization, including the carpet-like model, transient pore model, and inverted micelle model.^105^ However, in contrast to these passive transport mechanisms, CPP internalization has also been associated with endocytosis, an energy-dependent mechanism.^104^ Indeed, evidence suggests that CPPs are efficiently transported across the BBB via adsorptive-mediated transcytosis - a transport mechanism induced by electrostatic interactions between positively charged CPPs and the negatively charged luminal surface of ECs.^26,106^ Nevertheless, the exact mechanisms by which CPPs are translocated across the cell membrane remain poorly understood. Several uncertainties and controversies surround this topic, and several studies have found that CPPs may use multiple pathways instead of a single mechanism.^105,107,108^

Kang *et al.* used SIWV (Serine-Isoleucine-Tyrosine-Valine) peptide, a CPP from annexin-A3, attached to the surface of porous silicon nanoparticles (pSiNPs) loaded with 7-ethyl-10-hydroxycamptothecin (SN-38). This modification improved targeting and therapeutic efficacy in a GBM xenograft mouse model and demonstrated the feasibility of SIWV as a tumor-homing peptide for drug delivery to GBM.^109^ In another study, gold nanoparticles (AuNPs) modified with p28 peptide, a CPP derived from a redox protein secreted by *Pseudomonas aeruginosa*, demonstrated a preference for infiltrating GBM cells with a clear contrast over normal brain tissue. Additionally, *in vivo* studies have highlighted the ability of the p28 peptide to boost the effectiveness of TMZ when combined.^110^ Moreover, elastin-like polypeptide NPs were conjugated with the CPP octa-arginine (R8), which increased the uptake of NPs and facilitated their penetration into an *in vitro* 3D tumor model (spheroids grown from U87 human GBM cells).^111^

Balzeau *et al.* attached the CPP NFL-TBS.40–63 (YSSYSAPVSSSLSVRRSYSSSSGS), to the surface of PTX-loaded lipid nanocapsules (LNC). *In vivo* studies showed that when conjugated with this CPP, the NPs specifically targeted the tumor, improving drug delivery to tumor sites.^112^ Interestingly, this group also studied the effect of this peptide in GSCs. Nanocapsules functionalized with NFL decreased the proliferation and self-renewal capacity of GSCs.^113^ Moreover, *in vitro* studies revealed that liposomes modified with NFL peptide increased their ability to penetrate GBM cells after passing through the BTB, demonstrating the ability of NFL to cross brain ECs.^114^ Other strategies involved a responsive peptide to the tumor microenvironment. Zhao and colleagues developed pH-sensitive liposomes loaded with doxorubicin (DOX) and functionalized with H7K(R_2_)_2_ peptide, a pH-responsive CPP. This pH-sensitive strategy demonstrated a specific targeting effect and facilitated DOX release from liposomes in C6 and U87 glioma cells. Antitumor activity of stimuli-responsive functionalized liposomes was revealed both *in vitro* and *in vivo* experiments.^115^

### 2.2 Merging Forces: Synergistic Multiligand Approaches

Multiligand strategies, compared to single-ligand ones, hold promise for precise cell targeting and improved cellular uptake, reducing unpredictability and enhancing targeting efficiency.^27^ NPs, with their various surface functionalization options, allow for the development of multiligand functionalized nanomedicines designed for the sequential targeting of BBB, GBM cells, and GSCs (despite being less common). This offers a model for precise drug delivery directly to pathological sites within the brain.^116,117^ (Figure 1)

Martins *et al.* developed a BBB-stimuli responsive DTX-loaded PEG–PLGA NP conjugated with ANG2 and l-Histidine. This dual-functionalized PLGA-NP exploited ANG2 to bind to overexpressed LDLR in the BBB. Subsequently, the acidic pH during the endosomal BBB pathway cleaved ANG2 and exposed l-Histidine to target L-type amino acid transporter 1 (LAT1), the latter overexpressed in GBM cells. *In vitro* studies showed that this approach enhanced BBB penetration, increased GBM uptake, and induced higher cytotoxicity. Furthermore, *in vivo* studies in nude mice demonstrated that the median survival rate and the number of long-term survivors experienced a nearly half-fold rise.^118^ Similarly, Tian *et al.* designed a “smart” PTX-loaded polymeric micelle modified with (HE)_5_ peptide and (RG)_5_ CPP. (HE)_5_ peptide served as a pH-sensitive polyanionic peptide to mask the positive charge of (RG)_5_ CPP at a neutral pH. After internalization, the cationic peptide was activated, and the micelles were selectively triggered in response to the acidic pH of the tumor microenvironment. *In vivo* results showed an accumulation of these NPs in brain and GBM tissues, significantly reducing tumor size without severe toxicity to peripheral tissues.^119^ A different approach took advantage of the overexpression of matrix metalloproteinases. It developed poly(ethylene glycol)-poly(ε-caprolactone) (PEG–PCL) NPs modified with an activatable low-weight molecular protein (ALWMP). The positive charges of the LWMP peptide were concealed by the anionic peptide E10, which was linked through an MMP-2 cleavable linker. *In vitro* studies confirmed the capability of the activatable CPP to penetrate GBM cells, and analyses in nude mice revealed a significant increase in median survival in the ALWMP-treated group compared to the nonactivatable CPP-treated one.^120^

Yang and co-workers developed cationic liposomes coloaded with DTX and vascular endothelial growth factor (VEGF) small interfering RNA (siRNA) conjugated with ANG2 and tLyp-1 to improve BBB penetration and GBM targeting, demonstrating a superior efficacy against GBM.^121,122^ Another peptide, Ft peptide, was designed by combining tLyp-1 and FHK peptide (FHKHKSPALSPV) to target NRP-1 and tenascin C, respectively - both receptors overexpressed in GBM. Conjugation of Ft peptide with PTX-loaded PEG–PLA NPs significantly increased median survival time in nude mice compared to single functionalization with tLyp-1 and FHK peptide.^123^ Zhu *et al.* developed DTX-loaded nanomicelles cofunctionalized with ANG2 and TAT CPP, exhibiting enhanced BBB penetration, glioma cellular uptake, and accumulation. In an orthotopic U87 GBM-bearing mice model, the treatment showed prolonged blood-circulation time and significantly improved tumor inhibition compared to single-decorated controls. This resulted in an extended median lifespan with minimal side effects.^124^ In another work, ANG2 was combined with an activatable R8 CPP. After BBB penetration and GBM internalization mediated by the binding of ANG2 to LDLR, elevated levels of MMP-2 in the tumor site led to the activation of R8 CPP. Conjugation of these peptides with DTX-loaded PEG–PCL NPs demonstrated the most effective antiglioma activity in both *in vitro* and *in vivo* experiments.^125^ A tandem peptide involving the conjugation of R8 CPP with cRGD was attached to the surface of liposomes, revealing the capacity to cross the BBB and target glioma sites through a synergistic effect between both peptides.^126^ Studies in C6 stem cells also demonstrated the capacity of these tandem-modified liposomes in penetrating GSCs, mainly exacerbated by R8 CPP.^127^ Regarding tandem strategies, this group designed a different conjugation with R8 CPP and dGR peptide, a reverse sequence of RGD. This tandem approach formed a CendR motif, targeting integrin α_v_β_3_ and NRP-1 receptors. This dual receptor binding strategy revealed a triple targeting capacity of BBB, GBM cells, and GSCs, which more than doubled the median survival time of C6-bearing mice compared to the nontreated group.^128^ An arsenic trioxide-loaded poly(amidoamine) (PAMAM) dendrimer was modified with iRGD and TGN peptide (TGNYKALHPHNG), which was found to have shown a high capacity to penetrate the BBB. TGN was used as the first ligand to cross brain ECs, and iRGD acted as a second ligand to target GBM cells (integrin α_v_β_3_ and NRP-1 receptors). This strategy improved therapeutic efficacy and prolonged survival compared to single functionalization with each peptide.^129^ In another work, iRGD and SIWV CPP were combined and attached to TMZ-loaded pSiNPs, increasing penetration into deep GBM cells and higher anticancer efficacy.^130^

Shi *et al.* modified PTX-loaded liposomes with a TR peptide consisting of a combination of c(RGDfK) peptide and TH CPP. In this stimuli-responsive approach, the “inactive” peptide targeted the integrin receptors in the BBB, and further pH activation exposed the properties of TH CPP. *In vivo* results showed that TR-liposomes could better target GBM cells and eradicate GSCs, demonstrating increased median survival time in glioma-bearing mice.^131^ In addition, the c(RGDfK) peptide was combined with peptide-22, a specific ligand for LDLR, and attached to DOX-loaded liposomes. This dual-functionalized approach was able to cross the BBB/BTB and target GBM cells, with *in vivo* studies showing a significant improvement in mean survival time.^132^ Zhang *et al.* developed TMZ and vincristine-coloaded nanostructured lipid carriers decorated with RGD peptide and lactoferrin (Lf), a member of the Tf family whose receptor showed to be overexpressed in GBM cells. This strategy exhibited synergistic effects, increasing drug concentration within the GBM cells and indicating a clear efficacy in inhibiting tumor growth with reduced systemic toxicity.^133^ Gao and collaborators designed DTX-loaded PEG–PCL NPs functionalized with RGD and IL-13 peptides to target the BBB and GBM cells, respectively. *In vitro* results showed an enhanced cellular uptake and cytotoxicity, and *in vivo* experiments revealed an increased average survival period compared to single decorated NPs.^134,135^ A different study was explored, which decorated PTX-loaded PEG–PLGA NPs with Pep-1 and CREKA peptide (Cys-Arg-Glu-Lys-Ala). Pep-1 was used to penetrate the BTB and home NPs to GBM cells through IL-13Rα2, while the CREKA peptide was designed to target fibrin-fibronectin complexes, aiming to improve its retention in GBM. *In vivo* research verified that this dual modification led to enhanced NP accumulation and deeper penetration into GBM tissue, with an observed increase in median survival time.^136^ In a different approach, Lv *et al.* developed dual-functionalized PTX-loaded PEG–PLGA NPs with CGKRK peptide and Pep-1 to target heparan sulfate receptor in the BTB and IL-13Rα2 at GBM cells, respectively. *In vivo* studies demonstrated an extended median survival time associated with this strategy.^137^ Lakkadwala *et al.* designed 5-fluorouracil (5-FU)-loaded liposomes modified with Tf and penetratin (Pen) CPP, demonstrating higher biocompatibility and cellular uptake. This approach enhanced the concentration of 5-FU in the GBM cells, thereby exhibiting its efficacy in combating the tumor.^138^ The same group developed DOX and erlotinib (Erlo) coloaded liposomes modified with Tf and PFVYLI CPP, with *in vitro* studies showing higher cellular uptake enhanced anti-GBM activity.^139^ Furthermore, they formulated the same DOX and Erlo coloaded NPs but dual-functionalized with Tf and Pen CPP, exhibiting efficient penetration across the BBB and greater concentration of chemotherapy in GBM-bearing nude mice, resulting in a notable extension of survival duration.^140^ Moreover, Lakkadwala and co-workers also developed dual-modified liposomes coated with Tf and one of two CPPs, TAT or QLPVM. The combined use of Tf and CPPs in these liposomes resulted in a synergistic effect, facilitating interaction with the cell membrane and subsequent binding of Tf to its receptor.^141^ In another study, PEG–PLGA NPs loaded with palbociclib were decorated with T7 peptide and R9 CPP. *In vitro* studies showed that this dual-functionalization enhanced transport across the BBB and resulted in a more efficient inhibition of GBM cells.^142^ Zong *et al.* modified DOX-loaded liposomes with T7 peptide and TAT CPP; the latter was used to increase the penetration into the tumor. *In vivo* studies demonstrated an enhanced distribution within GBM areas, along with a notable extension in the improved median survival time of mice.^143^ Ying and co-workers designed a liposome dual-decorated with ^D^CDX and ^D^A7R (^D^R^D^P^D^P^D^L^D^W^D^T^D^A) peptide to target nAChRs in the BBB and both VEGFR-2 and NRP-1 receptors (in the BTB and GBM cells), respectively. The results from both *in vivo* and *in vitro* studies showed that this strategy enhanced the anti-GBM efficacy.^144^ In another work, a “Y-shaped” peptide named ^D^WVAP was designed to conjugate ^D^VAP peptide and ^D^WSW peptide, with affinity to GRP78 and quorum sensing receptor (QSR), respectively. This “Y-shaped” peptide exploited the overexpression of QSR in the BBB and the upregulation of GRP78 in BTB, GBM cells, and GSCs to achieve an all-stage targeting strategy.^145^ Farshbaf and colleagues developed bortezomib-loaded nanostructured lipid carriers dual-functionalized with D8 peptide and RI-VAP peptide to target nAChRs in the BBB and GRP78 (in both BTB and GBM cells), respectively. This dual-functionalized approach showed excellent selectivity *in vitro* and *in vivo.* Also, it prolonged the median lifespan and antitumor efficacy in mice bearing intracranial glioma.^146^ Basso *et al.* designed cationic lipid NPs coloaded with atorvastatin and curcumin. Hyaluronic acid (HA) was coupled to the previously formed NPs through an electrostatic interaction to take advantage of the overexpression of its receptors (e.g., CD44) in GBM, giving the NPs stealth properties and serving as a backbone for coupling peptide ligands. Lipid nanocarriers were further functionalized with c(RGDfK) peptide (to target both BBB and GBM cells) and H_7_K(R_2_)_2_ responsive CPP to also target tumor cells and promote a higher accumulation in the tumor. The antitumor efficacy was confirmed by magnetic resonance imaging in mice models bearing GBM. Indeed, mice administered with the free drug exhibited an 181% increase in tumor growth compared to those treated with functionalized lipid NPs.^147^

### 2.3 Harnessing External Stimuli

**Figure 2:**
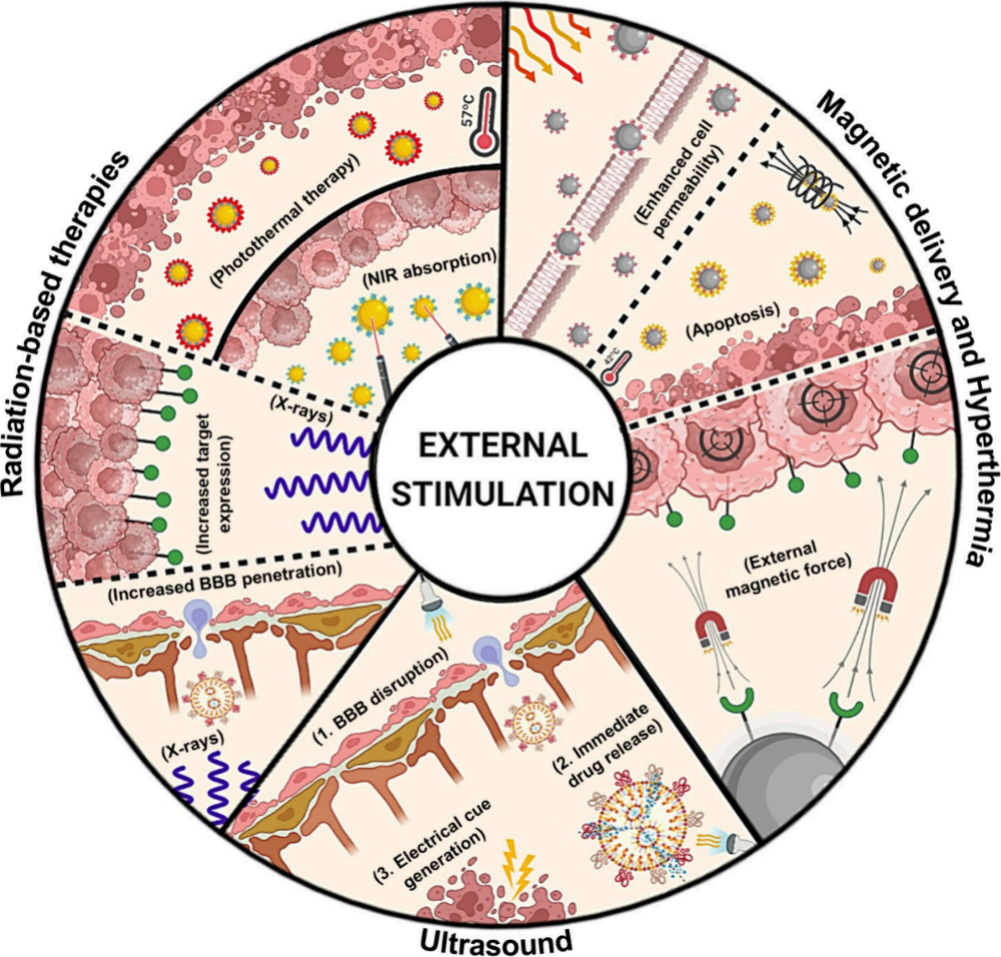
Innovative fusion: peptide surface modification meets external stimulation. Schematic representation of various external stimulation approaches—hyperthermia, photothermal therapy, magnetic delivery, ultrasound, and radiation therapy—that hold significant potential for enhancing the efficacy of GBM treatment regimens in combination with peptide functionalization. Abbreviations - BBB: Blood-brain barrier; NIR: Near-infrared spectroscopy. (Created with BioRender.com).

The complexity of GBM has motivated researchers to develop various therapeutic strategies beyond conventional ones. As noted by Wilhelm *et al.*, despite the significant advantages of active targeting over passive targeting, the efficacy of active targeting is less than 1%.^148^ Therefore, integrating external stimulation emerges as a strategic multimodal approach to improve antitumor efficacy, with peptides playing a pivotal role in enhancing GBM-targeted drug delivery. Combining external stimulation with peptide-mediated targeting aims to improve the precision and efficacy of drug delivery to GBM and address the inherent challenges associated with targeting this tumor. Indeed, approaches such as hyperthermia (HT), photothermal therapy (PTT), and the use of other external stimuli are being explored to improve GBM treatment.^149^ (Figure 2)

#### 2.3.1 Magnetic Delivery and Hyperthermia

Magnetic delivery is a common technique in drug delivery that utilizes magnetic fields to guide drug-carrying materials, often NPs, to specific locations within the body. This method combines therapeutic agents with magnetic materials, allowing for precise control over drug localization.^150^ Using magnetic forces, researchers aim to improve the control of drug release in terms of both timing and location; it helps minimize systemic side effects while increasing the concentration of therapeutic substances precisely at the intended site of action.^151−153^ To achieve effective targeting, magnetic NPs should exhibit strong magnetic susceptibility. Smaller sizes are preferable, as they enhance magnetic susceptibility and reduce the risk of systemic harm to the BBB.^154^

Therefore, a dual-targeting strategy was developed using magnetic targeting and T7 peptide to actively target magnetic PLGA NPs coloaded with curcumin and PTX. The *in vivo* results showed that none of the animals died during the 35-day experiment in the group treated with the combination of magnetic targeting and functionalized NPs. In comparison, the survival rates of the groups treated only with functionalized NPs (without a magnetic field) and free combination drugs were 83% and 67%, respectively. These results also demonstrated reduced adverse toxicities, proving the synergy between peptide and magnetic targeting.^155^ In another work, Dash *et al.* designed a magnetic nanocomposite loaded with DOX based on reduced graphene oxide (rGO). The rGO NPs were further conjugated with PEG and a gastrin-releasing peptide receptor (GRPR) peptide to harness the detected overexpression of this receptor in GBM cells. Following the administration, magnetic rGO NPs were guided magnetically and exposed to NIR laser irradiation, thereby magnetic delivery, PTT, and chemotherapy. This triple approach revealed improved results, showing a survival time of 29 days, compared to 25.5 days and 20 days for DOX plus magnetic and DOX alone delivery, respectively.^156^

Hyperthermia (HT) employs physical stimuli to trigger NPs, raising the temperature around the cells encircled by these NPs. It can be achieved through different methods and heating sources, such as laser, radiofrequency, ultrasound (US), or magnetic fields. In mild HT, the surrounding temperature rises to 42 and 45 °C after stimulation, activating the immune system and inhibiting tumor growth. The increase in temperature can result in the degradation of intracellular proteins triggered by the activation of defective proteins within the cell’s apoptosis pathways.^157,158^ HT can also enhance cell membrane permeability, potentially improving drug delivery into tumor cells.^158,159^ Although various sources of HT exist, magnetic fields are the most used in GBM. Magnetic hyperthermia therapy (MHT) is based on the principles of localized HT, which employs NPs and applies an alternating magnetic field (AMF) to generate heat.^160^ Magnetic NPs have garnered interest in HT because of their ability to generate heat when exposed to an external AMF.^161,162^ Iron oxide NPs are considered a preferred heating agent for MHT because of their size-related magnetic properties, ease of functionalization with targeting ligands, and minimal toxicity.^163^ The HT approach appears to have significant advantages when combined with low-dose chemotherapy.^159^

Exploiting the potential of these magnetically responsive NPs, Pucci *et al.* designed a lipid-based magnetic nanovector (LMNV) functionalized with ANG2. LMNV encapsulated supermagnetic iron oxide nanoparticles (SPIONPs) and nutlin-3a as a chemotherapy agent. A microfluidic model using human-derived cells was fabricated and employed to test the targeting efficiency and the BBB crossing abilities of these functionalized SPIONPs. This dynamic *in vitro* platform showed that this nanovector successfully accumulated in GBM cells and, upon stimulation with an AMF, induced apoptosis through the synergistic effect of MHT and chemotherapy.^164^ The group further evaluated the *in vivo* therapeutic potential of ANG2-modified LMNV loaded with TMZ. Studies in nude mice revealed the same successful accumulation in GBM cells without exhibiting toxic effects. The synergy between MHT and chemotherapy suppressed tumor increased median survival time to 68 days, compared to 46 days and 42 days for modified NPs without MHT and control, respectively.^165^ Zhou *et al.* developed PEGylated Fe@Fe_3_O_4_ NPs decorated with c(RGDyK) peptide. Through the specific interaction between integrin α_v_β_3_ protein and c(RGDyK) peptide, coupled with the MHT properties under AMF, the NPs exhibited an outstanding efficacy in tumor ablation. Indeed, on the 15th day, the tumor volumes in the saline group, the saline + AMF group, and the functionalized NPs group increased to approximately 13.7, 10.8, and 8.5 times their initial sizes, respectively. In contrast, the tumor volumes in the MHT + functionalized NPs group were effectively reduced by half.^166^ In a different approach, Senturk and colleagues developed curcumin-loaded SPIONPs coated with PLGA-*b*-PEG diblock copolymer. These SPIONPs were conjugated with the Glycine-Arginine-Glycine-Aspartic acid-serine (GRGDS) peptide, with an affinity for the α_v_β_3_/α_v_β_5_ integrins, thereby targeting them more effectively to GBM cells. MHT was performed using a radiofrequency magnetic field, a specific type of AMF. *In vitro* results showed that GRGDS peptide conjugated NPs significantly increased the bioavailability of curcumin and reduced the required therapeutic dose by up to 6-fold compared to nonconjugated NPs. Moreover, when combined with MHT, the results indicated that hyperthermia could enhance NP uptake through nonthermal mechanisms. Further studies are needed to understand the mechanisms behind this. Still, overall, the data showed that the combination of GRGDS-functionalized NPs with MHT could be useful for targeted delivery of curcumin to GBM cells.^167^

#### 2.3.2 Radiation-Based Therapies

##### 2.3.2.1 Photothermal Therapy

Photothermal therapy (PTT) is a therapeutic technique commonly used in cancer treatment in which light-absorbing agents absorb specific wavelengths of light, typically in the near-infrared (NIR) region. These agents convert the absorbed light into heat, resulting in localized hyperthermia in the target tissue. This localized heating induces cell death in tumor cells, making PTT a valuable tool in the fight against GBM.^168,169^ Since most photosensitizers and photothermal agents are hydrophobic and have low tumor selectivity, nanotechnology plays a vital role in PTT.^149^ NPs, such as AuNPs or iron oxide NPs, are irradiated with NIR light, which is absorbed and converted into localized heat, resulting in the precise destruction of GBM cells within a typical temperature range of 50 to 60 °C.^170^

Wu *et al.* designed polydopamine (PDA)-NPs, well-known for their photothermal conversion and biocompatibility, loaded with TMZ and modified with Pep-1 to target IL-13Ra2 overexpressed in both BBB and GBM cells. When exposed to an 808 nm NIR laser, the PDA-NPs generated heat, activating the PTT effects. *In vivo* studies demonstrated that this combination of targeted chemotherapy and PTT resulted in significantly improved antitumor efficacy compared to the use of chemotherapy alone. This suggests that the dual approach of combining PTT with chemotherapy could be more effective in treating GBM.^171^ In another study, self-assembled pH-responsive NPs loaded with modified camptothecin (CPT) and functionalized with ANG2 were also irradiated with an 808 nm laser. The *in vivo* results in nude mice outlined a similar synergy between PTT and chemotherapy, with this combined approach exhibiting excellent tumor ablation and reduced side effects. Indeed, the median lifespan of glioma-bearing mice treated with ANG2-modified NPs plus PTT was the highest (60 days) compared to ANG2 functionalized NPs without PTT (51 days), nonfunctionalized NPs with PTT (33 days), and nonfunctionalized NPs without PTT (30 days).^172^

He and co-workers devised an innovative nanosystem modified with c(RGDfK) peptide. These NPs incorporate an intense electron donor, dithienopyrrole (DTP), and a strong electron acceptor, thiadiazol benzotriazole (TBZ), originating from a near-infrared II (NIR-II) optical absorptive conjugated polymer (PDTP-TBZ). In this context, PDTP-TBZ NPs were irradiated to initiate photothermal effects. *In vivo* experiments revealed that modified NPs irradiated with NIR-II had a significant antitumor effect, effectively targeting and destroying GBM cells with reduced toxicity. In contrast, tumor proliferation was only slightly inhibited by functionalized NPs without irradiation. In addition, when NIR-II irradiation was used as a control, tumor growth was not inhibited.^173^ Another strategy is the use of gold nanorods to promote PTT. Gonçalves *et al.* synthesized gold nanorods functionalized with a nestin-binding peptide to target nestin proteins specifically overexpressed on GSCs. This targeting approach resulted in the specific destruction of GSCs through the induction of cell apoptosis. In contrast, cells that did not express nestin remained unharmed and viable.^174^ In another work involving magnetic NPs, Zhou *et al.* presented a distinct approach for the PEGylated Fe@Fe_3_O_4_ NPs modified with c(RGDyK) peptide. In addition to evaluating the photothermal properties, the in vivo performance of NPs, including biodistribution and pharmacokinetics, was assessed, and these demonstrated excellent targeting properties. This system has been proven to be highly effective for targeted PTT. The study found that the tumor volumes of the group treated with laser only and the group treated with functionalized NPs were approximately 8 times larger on the last day (after 15 days) compared to the first day. However, the tumor in the group treated with PTT plus modified NPs had nearly disappeared by day 8.5.^175^

##### 2.3.2.2 Radiation Therapy

Radiotherapy utilizes ionizing radiation, unlike PTT, which employs nonionizing radiation.^176^ While RT has traditionally been part of the primary treatment for cancer, as it is used in the Stupp protocol, it is associated with some limitations and side effects. A promising alternative is emerging through the combination of radiotherapy with NPs. It increases the sensitivity of tumor cells to radiation, enabling targeted treatment and reducing side effects on healthy tissues.^4,177^ RT has been proven to effectively increase the accumulation of NPs in tumors and improve their intratumoral distribution through short treatment sessions.^178^ Tamborini and colleagues investigated the potential synergy between RT and functionalized NPs. For this purpose, they used PLGA NPs modified with the CTX peptide, taking advantage of their binding properties to MMP-2 and ClC-3. Thus, they demonstrated that X-ray irradiation increased BBB permeabilization and enhanced the expression of the CTX targets. This proved to be a promising strategy for improving therapeutic cargo on GBM cells, as the binding of CTX-NPs with MMP-2 reduced its catalytic activity by 50%.^179^ Furthermore, Erel-Akbaba *et al.* developed SLNs functionalized with cyclic iRGD peptide for a combined gene delivery immunotherapy against EGFR and programmed cell death ligand-1 (PD-L1), both contributing to tumor development and proliferation. When combined with short bursts of RT, this approach improved the targeting efficiency of NPs, resulting in enhanced downregulation of EGFR and PD-L1 and increased tumor growth inhibition. Moreover, *in vivo* studies showed that mouse survival was significantly higher (38 days) with the combination of RT and modified SLNs compared to the RT plus nontargeted group (24.5 days), functionalized SLNs without RT (24 days) and the control group (21 days).^180^

#### 2.3.3 Ultrasound

Focused ultrasound (FUS) technology employs high-frequency sound waves to create either thermal or mechanical effects in specific tissues within the body. These ultrasound waves are precisely directed toward a particular point, enabling them to induce various therapeutic outcomes.^181^ FUS has gained recognition as a noninvasive technique for opening the BBB through different mechanisms like thermal effects, the disruption of endothelial tight junctions, or cavitation.^182^ Indeed, DOX-loaded liposomes were conjugated with a designed atherosclerotic plaque-specific peptide-1 (AP-1) to target the overexpressed interleukin-4 receptors (IL-4R) in GBM cells. The application of FUS increased the accumulation of DOX in tumor cells, demonstrating the efficacy of this method in disrupting the BBB and achieving high chemotherapy doses at minimal systemic toxicity. *In vivo* studies showed that combining FUS with unconjugated liposomes and AP1-modified liposomes increased the concentration of DOX in the tumor by 147% and 202%, respectively.^183,184^

FUS has also been shown to be effective in the induction of drug release. ANG2-modified PLGA hybrid NPs, coloaded with DOX/perfluorooctyl bromide, exhibited burst release at GBM sites upon US irradiation, delivering almost 50% of the cargo within 2 min. *In vivo* results demonstrated that combining functionalized NPs and US irradiation increased the median lifespan of the GBM-bearing mouse model to 56 days, compared to 37.5 days and 17 days for functionalized NPs without US irradiation and the control group, respectively.^185^ Besides, US techniques can be applied in the treatment of GBM to generate anticancer cues. In this context, Pucci and co-workers developed piezoelectric hybrid lipid-polymeric NPs functionalized with ApoE and loaded with nutlin-3a, a nongenotoxic drug. US stimulations triggered drug release and a cellular response through the activation of calcium channels. This demonstrated the generation of an effective anticancer electrical cue, presenting the ability to reduce the invasiveness of T98G cells while promoting necrotic and apoptotic events.^186^

### 2.4 Exploring Biomimetic Techniques

Biomimetic nanocarriers are NPs designed to mimic the properties and functions of natural biological structures.^187^ Due to their inherent biointerfacing capacity, they have garnered significant attention in the past few years.^188^ Similarly to applying external stimuli, integrating biomimetic approaches with peptide-guided targeting is expected to improve the precision and effectiveness of targeted drug delivery to GBM, addressing the fundamental barriers to targeting this tumor type.

#### 2.4.1 Cell Membrane and Endogenous Protein Camouflage

**Figure 3:**
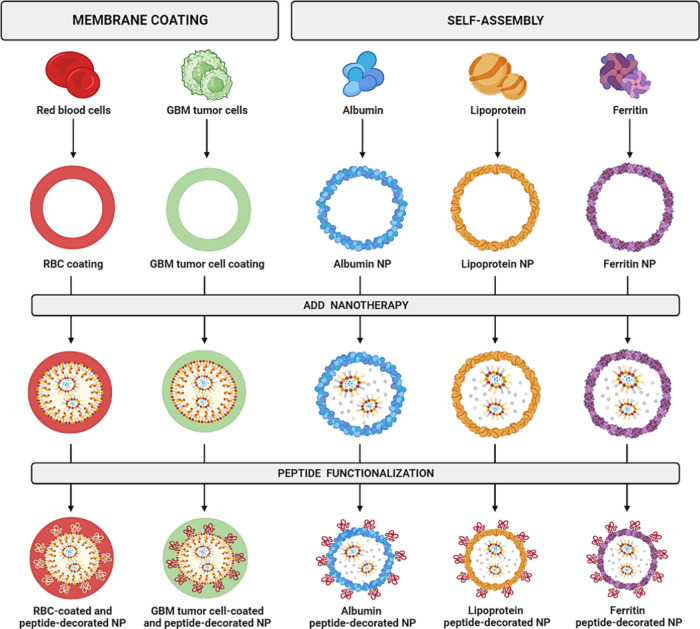
Cell membrane and endogenous protein camouflaged nanocarriers. This figure illustrates two prominent biomimetic approaches in decorated nanocarrier development for GBM treatment: membrane coating and self-assembly. Membrane coating involves RBC and GBM tumor cell membranes, imparting natural biological properties to nanocarriers for enhanced targeting and reduced immunogenicity. On the other hand, self-assembly uses serum albumin, lipoprotein, and ferritin to create nanocarriers with inherent structural advantages, optimizing therapeutic delivery to specific targets within the challenging GBM microenvironment. Abbreviations: GBM: Glioblastoma; NP: Nanoparticle; RBC: Red blood cell. (Created with BioRender.com)

There has been significant interest in cell membrane-based biomimetic nanotechnology vehicles (Figure 3), which take advantage of natural cell membranes and synthetic NPs and exhibit a better targeting ability with enhanced cell compatibility, prolonged circulation in the body, and minimal activation of the immune response.^189,190^ Indeed, this technology is a promising strategy against GBM as it may enhance stealth characteristics, reducing macrophage recognition and further *in vivo* elimination.^191^

The pioneering work that used cell membrane-coating NPs to target GBM involved coating PLGA NPs with red blood cell membranes (RBCm) modified with the ^D^CDX peptide, revealing lower systemic toxicity and improved therapeutic efficacy. *In vivo* studies conducted on U87 glioma-bearing mice showed that RBCm-coated NPs functionalized with DCDX peptide significantly increased the survival rate of mice to 28.5 days, compared to RBCm-coated NPs without ^D^CDX functionalization (23.5 days) and the control group (22 days).^192^ Recently, Liu *et al.* designed a RBCm-camouflaged NP decorated with ApoE-peptide for the selective targeting of mRNA to GBM cells. To facilitate the intracellular delivery of mRNA, a charge conversion strategy was employed, triggered by the slightly acidic environment of the endosome, leading to the disruption of the erythrocyte structure. *In vitro* studies demonstrated a 2.5-fold increase in the uptake of functionalized RBCm-coated NPs compared to nonfunctionalized ones. Additionally, *in vivo* studies showed a significantly longer median survival time for Apo-E functionalized biomimetic NPs (49 days) compared to nonfunctionalized (31 days) and the control group (23 days).^30^ This strategy had previously been used by the same group in the development of siRNA-loaded RBCm NPs functionalized with ANG2.^193^ Additionally, they used an ApoE functionalized RBCm platform for the codelivery of TMZ and a bromodomain inhibitor.^194^ They all showed BBB penetration, tumor accumulation, and improved pharmacokinetics without negligible side effects.^30,193,194^ In addition, the same team proposed two similar approaches utilizing RBCm NPs: one was modified with ApoE for the codelivery of antiapoptotic protein inhibitors, and another functionalized with ANG2 and loaded with DOX and lexiscan. While mechanistically similar to earlier procedures, these two latter approaches are distinguished by the presence of a pH-sensitive dextran inner core responsible for the burst release of the molecules.^31,195^ Chai and co-workers developed DTX-loaded nanocrystals coated with RBCm, subsequently modified with the c(RGDyK) peptide to target integrins. The RBCm stabilized the nanocrystals, enhancing biocompatibility and reducing side effects. The peptide functionalization resulted in increased DTX accumulation in tumor sites, boosting their effectiveness both in a subcutaneous U87 model and in orthotropic U87 glioma-bearing mice. In the mice orthotopic model, c(RGDyK) modified RBCm coated NPs significantly prolonged the median survival time (62 days) compared to nonfunctionalized biomimetic NPs (34.5 days) and the control group (32 days).^196^ Beyond erythrocyte membrane strategies, Wang and co-workers developed a nanomedicine cloaked with GBM cancer cells to take advantage of the homotypic binding mechanism of these membranes. These NPs were further decorated with an ApoE peptide for the codelivery of TMZ and lomeguatrib - a MGMT inhibitor. This strategy revealed a higher capacity to cross the BBB, enhancing sensitivity to chemotherapy resistance and GSCs. *In vivo* experiments conducted on orthotopic U251-TR-bearing mice showed a significant reduction in tumor growth.^197^

In addition to cell membrane coating techniques, several endogenous proteins with biomimetic attributes can also be used as efficient carriers for drug delivery (Figure 3), leveraging their inherent nonimmunogenic and nontoxic properties.^198^ Therefore, Zhao *et al.* developed an albumin-based carrier that could target overexpressed proteins in the tumor cells *per se* and further functionalized with TfR-T12 peptide to enhance BBB penetration and tumor cell uptake. Both *in vitro* and *in vivo* studies showed efficient inhibition of glioma cell proliferation with an excellent treatment outcome. Indeed, results from an orthotopic glioma-bearing model showed that nanocarriers functionalized with TfR-T12 peptide had the most extended median lifespan (42 days) compared to nonfunctionalized nanocarriers (28 days) and the saline group (24 days).^199^ A ferritin nanocage coloaded with epirubicin (EPI) and CPT, possessing inherent TfR targeting capabilities, was further enhanced by attaching RGD peptides to its outer surface. This design enabled dual targeting of CD71 and integrin α_v_β_3_, thereby enhancing selectivity. The spatiotemporally programmed cascade release of EPI and CPT prolonged their retention time and increased their anti-GBM activity. *In vivo* results showed that these nanocages inhibited the growth of orthotopic glioma with minimal toxicity, leading to a prolonged median survival time for mice. The observed results were attributed to their improved capability to penetrate the BBB, specifically target GBM cells and the synergistic action of the combined drugs.^200^

Moreover, Geng *et al.* developed biomimetic NPs coloaded with TMZ and salinomycin. These NPs, based on low-density lipoprotein (LDL) with apolipoprotein B (ApoB) as the outermost layer, were further decorated with ANG2 to synergistically target LDLR and LRP-1, respectively. *In vitro* studies showed that the active targeting effect of the nanocarriers increased drug accumulation, resulting in significant inhibition of GSCs, mainly attributed to the effect of salinomycin (an anti-GSC drug). *In vivo* studies in glioma-bearing mice demonstrated an increased lifespan of these biomimetic carriers compared to controls, with low immunogenicity and no significant toxicity. These findings displayed the potential of this biomimetic functionalized vehicle for GBM therapy.^201^

#### 2.4.2 Extracellular Vesicle-Based Nanosystems

Extracellular vesicles (EVs) are lipid-bound vesicles released into the extracellular space by all types of cells. Based on their origin, size, secretion mechanism, function, and composition, EVs can be classified into three major subtypes: exosomes (30–150 nm), microvesicles/ectosomes (50 nm-1 μm), and apoptotic bodies (50 nm-5 μm).^202−204^ The intrinsic biocompatibility, biodegradability, low toxicity, and nonimmunogenic nature of EVs surpass traditional nanomaterials, making them ideal for drug delivery. Their lipid bilayer protects the cargo from degradation, thereby increasing stability in the circulation. In addition, EVs can evade the immune system and penetrate physiological barriers.^205^ After systemic administration, most unmodified EVs have limited ability to reach the brain. Therefore, modifying EVs is essential to fully realize their potential as a nov delivery system to the brain. This includes engineering techniques such as peptide functionalization to enhance their targeting and penetration capabilities.^206^

Liu *et al.* designed a complex hybrid exosome nanocarrier. Hydroxychloroquine (HCQ)-loaded hollow zinc sulfide (ZnS) NPs were coated with exosomes derived from human U87 GBM spheroids. These exosome-coated NPs were then fused with redox- and pH-responsive iRGD-modified liposomes. This hybrid design was intended to take advantage of the targeting ability of iRGD peptide to cross the BBB and reach the tumor, as well as the homing ability of exosomes to GBM cells. The exosome membrane was then exposed to fuse with GBM cells due to high glutathione levels in the acidic tumor microenvironment. Both *in vitro* and *in vivo* studies showed a selective and efficient accumulation in GBM cells, leading to the accumulation of HCQ within lysosomes with minimal toxicity. In fact, the median lifespan of mice treated with functionalized exosomes (54 days) was higher than that of unfunctionalized exosomes and free HCQ, which had median survival times of 42.5 days and 30 days, respectively.^207^ In another work, Wang and co-workers developed exosome-liposome hybrid nanovesicles coloaded with traditional Chinese medicine cryptotanshinone and salvianolic acid B. These hybrid nanovesicles were developed by membrane fusion between blood exosomes and tLyp-1 peptide-modified liposomes. Blood exosomes highly expressed TfR receptors on their surface and could adsorb Tf in blood. Therefore, this dual engineering modification of TfR and tLyp-1 peptide enhanced targeting across the BBB and GBM cells. In addition, the membrane component of the exosomes endowed the hybrid nanovesicles with the ability to evade phagocytosis by the immune system.^208^

Liu *et al.* engineered artificial EVs from embryonic kidney 293T (HEK293T) cells loaded with DOX. ANG2 was anchored to the surface of these EVs using the synthetic peptide TRP-PK1 (AYLAVMVFALVLGWMNALYFTRGL). A DNA clone expressing the ANG-TRP-PK1 fusion peptide was transfected into HEK293T cells for expression and presentation of the fusion peptide in the cell membrane. DOX-loaded cells (after electroporation) were further squeezed with a liposome extruder to create peptide-functionalized artificial EVs for targeted delivery. These engineered DOX-loaded artificial vesicles demonstrated high BBB penetration and GBM cell targeting ability with minimal side effects. *In vivo* studies in an orthotopic U87 GBM-bearing mice model demonstrated effective tumor suppression and significantly improved survival and lifespan of the mice.^209^ Zhou *et al.* developed GBM cell-derived exosomes coloaded with TMZ and DOX. These nanosystems were further decorated with ANG2 to target the BBB and GBM cells and with CD133 (WRLRWHSPLKGGC) peptide to target GSCs. Both *in vitro* and *in vivo* results showed high crossing ability across the BBB and GBM cells, as well as deep penetration into the tumor parenchyma. Furthermore, *in vivo* studies in orthotopic syngeneic GBM-bearing mice extended their survival.^210^ Zhu and colleagues developed small DOX-loaded EVs and dual-functionalized them with ANG2 to target both the BBB and GBM cells and TAT-CPP to target GBM cells and overcome ANG2 receptor saturation. The high efficiency of this system in penetrating the BBB and tumor cells was evaluated *in vitro* and revalidated in orthotopic glioma mice models. *In vivo* studies showed a 2-fold survival of glioma mice compared to nonfunctionalized EVs, with few side effects.^211^

Lee and his research group developed two different approaches using the T7 peptide.^212,213^ In the first, they produced modified exosomes loaded with antisense miRNA oligonucleotides against miR-21 (AMO-21). Peptide decoration was achieved by incorporating T7 into the exosome membrane as a fusion protein of T7 and Lamp2b.^212^ More recently, this group used cell membrane nanovesicles (CMNVs) loaded with AMO-21. These CMNVs were prepared by extrusion of C6 cell membrane fragments to mimic exosomes and further functionalized with cholesterol-conjugated T7 (T7c) peptide (T7c) through hydrophobic interaction.^212,213^ Both approaches showed promise for GBM gene therapy. However, the latter suggested potential advantages in terms of stability, lower toxicity, and clinical translation (due to the ease of large-scale efficient production).^212,213^

Geng *et al.* functionalized small EVs with cyclic arginine-glycine-aspartic acid-tyrosine-cysteine (c(RGDyC)) peptide to target integrin α_v_β_3,_ overexpressed in both the BBB and GBM cells. The surface modification was accomplished through a two-step reaction: first, hydrophobic attachment of a PEGylated lipid followed by chemical binding of the c(RGDyC) peptide to the terminal end of the PEG. *In vitro* studies revealed a 2.4-fold increase in cellular uptake and a 1.7-fold increase in DOX delivery efficiency of functionalized small EVs compared to nonfunctionalized small EVs.^214^ In another study, Zhu and colleagues modified PTX-loaded exosomes secreted by embryonic stem cells with c(RGDyK) peptide. This modification was also intended to target the α_v_β_3_ integrin receptors in the BBB and GBM cells. Both *in vitro* and *in vivo* models confirmed the inhibitory effect of nonfunctionalized exosomes on GBM. Thus, it was shown that the effectiveness of PTX was enhanced through targeted functionalization, as demonstrated by an *in vitro* GBM model and *in vivo* subcutaneous and orthotopic xenograft models.^215^ Jia *et al.* coloaded SPIONs and curcumin into exosomes by electroporation. They then decorated the exosome membrane with the RGE (RGERPPR) peptide, a specific ligand of the NRP-1 receptor, using click chemistry. The engineered exosomes exhibited excellent stability and biocompatibility. Furthermore, *in vitro* and *in vivo* studies demonstrated efficient BBB crossing and promising results for targeted imaging and GBM therapy. The median survival time of RGE-modified exosomes (65 days) was significantly higher than that of nonfunctionalized (52 days), free curcumin (35 days), and free SPIONs (33 days).^216^

### 2.5 Pushing Boundaries: What Can Nanocatalytic Techniques Bring Us?

A concept that combines nanomedicine and nanocatalysis has arisen in the previous few years: “nanocatalytic medicine”.^217^ Catalytic therapy triggers precise reactions *in situ* with minimal toxicity in response to specific signals from the tumor environment or external stimuli.^218^ Specifically designed to facilitate catalytic effects within the affected areas, nanomaterials reduce the energy required for these reactions and effectively generate reactive oxygen species (ROS), enabling a variety of dynamic treatments in diseased tissues.^219,220^ This approach offers significant advantages over conventional chemotherapy, delivering more effective therapeutic outcomes in tumor elimination and fewer side effects.^221,222^

**Figure 4:**
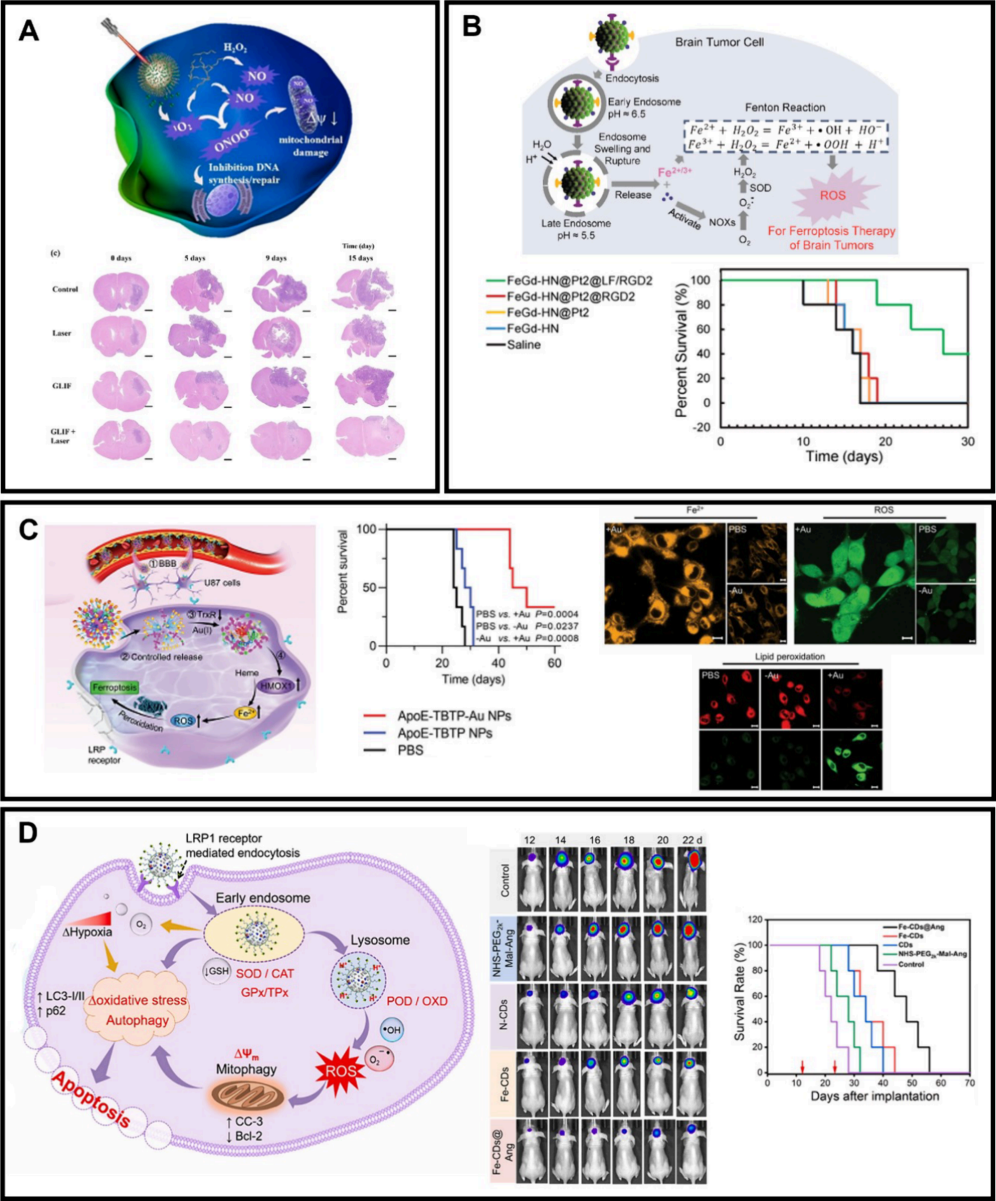
(A) Illustration of GLIF mechanism in GL261 cells. H&E staining to brain slices of tumor-bearing mice. Adapted with permission from ref.^223^ Copyright 2023, Elsevier (B) Graphical representation for the ferroptosis therapy of brain tumors with cisplatin-loaded Fe_3_O_4_/Gd_2_O_3_ NPs decorated with Lf peptide and RGD2. *In vivo* results of treated mice bearing orthotopic brain tumors. Adapted with permission from ref.^227^ Copyright 2018, American Chemical Society (C) Schematic representation of ApoE-TBTP-Au NPs for ferroptosis therapy in orthotopic GBM-bearing mice model via thioredoxin reductase-HMOX1 axis. *In vivo* results of the tumor-bearing mice. Confocal images of labile iron (Fe^2+^), ROS, and lipid peroxidation in U87 cells stained with FerroOrange, DCFH-DA, and BODIPY probes, respectively. Adapted with permission under a Creative Commons CC BY license from ref.^229^ Copyright 2023, John Wiley and Sons (D) Schematic illustration of the enzymatic cascade initiated by ANG2-modified FeCDs nanozymes. *In vivo* efficacy in an orthotopic U87MG-Luc tumor-bearing nude mice. Adapted with permission from ref.^232^ Copyright 2022, Elsevier.

Xue *et al.* exploited the use of a mesoporous SiO_2_ template to prepare Gd_2_(WO_4_)_3_:Nd^3+^ NPs and then constructed a multifunctional nanoagent named “GLIF”. These NPs were designed by loading poly-l-arginine (PLA) and indocyanine green (ICG) – photosensitizer - on the surface. Lf was attached to enhance the targeting ability in GBM, taking advantage of the overexpression receptor in GBM cells. Under 808 nm excitation, ICG generated singlet oxygen (^1^O_2_), serving as a photosensitizer and activating PLA for increased nitric oxide (NO) generation. Moreover, ^1^O_2_ from ICG and NO from PLA could switch to peroxynitrite (ONOO^–^), which has a longer lifetime and higher toxicity than ROS, resulting in potent tumor-killing effects. This strategy effectively inhibited GL261 cell migration and impaired DNA synthesis mitochondrial function, which proved to be promising for GBM therapy.^223^ (Figure 4A).

Ferroptosis, a nonapoptotic type of cell death, typically involves significant iron accumulation and lipid peroxidation as part of the cell death mechanism.^224,225^ In this context of ferroptosis, the Fenton reaction–comprising the interaction of iron (II or III) with hydrogen peroxide (H_2_O_2_) to produce ROS - plays a crucial role. Consequently, various nanomaterials have been specifically engineered for cancer therapy based on ferroptosis.^226^

Shen *et al.* developed cisplatin-loaded Fe_3_O_4_/Gd_2_O_3_ NPs decorated with Lf and RGD peptide dimer (RGD2) to target Lf receptors in the BBB and integrin α_v_β_3_ (RGD2 receptor) in GBM cells, respectively. Fe_3_O_4_/Gd_2_O_3_ NPs released Fe^2+^, Fe^3+^, and cisplatin upon internalization, providing two of the three components required for the Fenton reaction. Furthermore, the released cisplatin indirectly produced H_2_O_2_, prompting the Fenton reaction. This cascade resulted in the generation of ROS, inducing the GBM cell death. *In vivo* studies conducted in orthotopic GBM-bearing models demonstrated the successful inhibition of tumor growth with no evidence of toxicity. Mice treated with the dual-functionalized NPs showed a significantly longer median lifespan of 27 days, compared to 17 days for mice treated with nonfunctionalized NPs and 16 days for the saline groups.^227^ (Figure 4B).

In the context of noncanonical ferroptosis, characterized by increased activation of heme oxygenase-1 (HMOX1) and subsequent higher amount of labile iron pools, labile iron directly participates in the Fenton reaction, catalyzing the formation of free radicals and inducing lipid peroxidation.^228^ Based on these concepts, Zhang and co-workers designed TBTP-Au NPs to act as NIR-II ferroptosis activators. Modified with ApoE peptide, these TBTP-Au NPs selectively targeted and induced GBM cell death through controlled release triggered by the overproduced ROS in the tumor microenvironment. Within this microenvironment, the NPs selectively bound to overexpressed thioredoxin reductase and specifically activated HMOX1-regulated ferroptosis pathways. The application of NIR-II imaging to monitor tumor accumulation of NPs alongside fluorescence intensity quantification revealed that functionalized NPs exhibited a significantly enhanced ability to penetrate the BBB and accumulate in tumors compared to nonfunctionalized NPs. Moreover, the *in vivo* results showed a significant extension in the median lifespan of the orthotopic GBM-bearing mice model.^229^ (Figure 4C).

Nanozymes, characterized by their affordability, robustness, and enzyme-mimicking characteristics, have effectively initiated catalytic processes within tumor cells.^230,231^ The nanozymes currently employed in medical applications primarily mimic oxidoreductase enzymes. These nanozymes can be broadly categorized into four principal types based on their distinct catalytic functions: (i) peroxidase (POD), (ii) oxidase (OXD), (iii) superoxide dismutase (SOD), and (iv) catalase (CAT).^231^

Therefore, Muhammad *et al.* developed an ultrasmall Fedoped carbon dots (Fe-CDs) nanozyme, functionalized with ANG2 to target the overexpressed LRP-1 and accumulate in brain tumor areas. This nanozyme exhibited multifunctional enzymatic activities, including POD, OXD, SOD, and CAT, allowing for specific ROS regulation within the tumor microenvironment. The nanoenzyme induced significant tumor regression in GBM xenograft mouse models. Once accumulated in the acidic environment of endosome-lysosome, the nanozymes exhibited their inherent OXD/POD-like activities, impairing lysosomal degradation and activating autophagic flux. Additionally, their SOD, CAT, and glutathione peroxidase (GPx)-like activities regulated ROS, enhancing autophagy and lysosome-based apoptosis. Recognizing the role of hypoxia in coregulating autophagy, the activation of autophagy pathways by these nanozymes helped alleviate hypoxia, disrupting redox homeostasis and intensifying apoptotic cell death in GBM cells. *In vivo* results in an orthotopic U87 GBM-bearing mouse model showed that ANG2 functionalized NPs significantly increased median survival to 56 days, compared to less than 35 days for nonfunctionalized NPs and less than 30 days for the control group. This functionalized approach also revealed reduced systemic side effects.^232^ (Figure 4D).

Mansur and collaborators hypothesized the design of a hybrid nanosystem comprising two nanozymes. In this design, cobalt-doped magnetite and Au-NPs loaded in a carboxymethylcellulose organic shell were functionalized with iRGD peptide to enhance the targeting for a biocatalytic hyperthermal chemodynamic therapy of GBM cells. The catalytic reaction was based on the cascade reaction triggered by the Au-NPs, using glucose to produce H_2_O_2_. This H_2_O_2_, in turn, served as a substrate for generating highly oxidizing reactive radicals by cobalt-doped magnetite through Fenton-like reactions. The subsequent heat-induced cell death depended on the MHT of cobalt-dopes magnetite nanozymes when exposed to an external AMF. *In vitro* results showed that iRGD functionalized NPs exhibited a more pronounced cell-killing activity of approximately 60% against cancer cells compared to healthy cells. The functionalized nanosystem increased the cell-killing activity against U87 cancer cells by approximately 36% compared to the nonfunctionalized one. Moreover, the application of MHT to functionalized NPs revealed a 65% decrease in cell viability compared to the group of functionalized NPs without MHT.^233^

In a different study, Au-Clusters, stable transitional substances bridging the gap between large NPs and atoms, were decorated with a small peptide to target integrin α_ν_β_3_. Upon internalization, the functionalized Au-Clusters were catalysts, converting H_2_O_2_ into superoxide anion radicals (O_2_^–•^). This reaction notably increased the ROS levels in GBM cells, activating the mitochondrial apoptosis pathway, releasing caspases, and ultimately inducing apoptosis.^234^

Recently, there has been increasing interest in using ZnS for photodynamic therapy due to its light-responsive solid activity. Hollow ZnS NPs induce ROS through two bands: the valence band and the conduction band. The valence band generates hydroxyl radicals (^•^OH) by reacting with H_2_O, while the conduction band initiates a reduction process that generates ^1^O_2_ radicals by reacting with O_2_.^235^ The aforementioned functionalized hybrid exosomes developed by Liu *et al.* were also designed to take advantage of these photodynamic properties of ZnS. Therefore, a visible light source was used in this strategy to induce ROS from hollow ZnS NPs and damage GBM cells. HCQ was used to suppress autophagy activity and further enhance the cytotoxicity of ROS. The mice receiving iRGD-modified HCQ-loaded ZnS NPs and light illumination had a median survival of 73 days, which was significantly higher than all other groups.^207^

### 2.6 Bypassing the Blood–Brain Barrier: Alternative Routes of Administration

**Figure 5:**
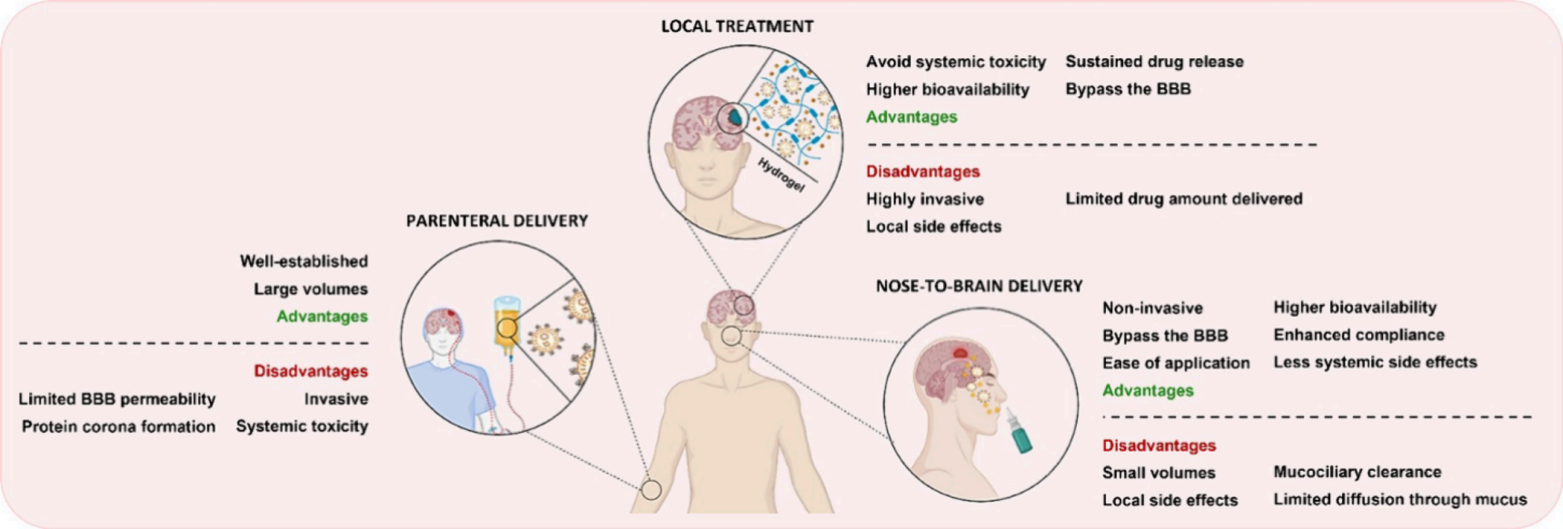
Delivery routes for targeted delivery in GBM treatment. (Created with BioRender.com).

Shifting the focus from engineering peptide NPs to cross the BBB to exploring alternative administration paths for brain delivery acknowledges the complex challenge that this barrier poses for drug delivery. Here, we discuss alternative delivery routes that use peptide-functionalized NPs or hydrogels to bypass the BBB and potentially improve the efficiency and specificity of brain delivery and GBM treatment. (Figure 5)

#### 2.6.1 Nose-to-Brain Delivery

Intranasal (IN) administration or nose-to-brain delivery is a promising alternative to the traditional parenteral route that allows the direct delivery of drugs from the nose to the brain, bypassing the BBB and reducing systemic side effects.^236,237^ Due to the direct link between the nasal cavity and the brain via the olfactory epithelium, the IN route of administration is the only pathway connecting the brain directly to the external environment.^237^ The nasal cavity has unique anatomical characteristics that make it an ideal route for minimally invasive drug delivery. Its large surface area and high-density microvasculature facilitate drug absorption and distribution.^238^ Direct drug transport from the nasal cavity to the brain occurs via two main routes: the trigeminal and the olfactory pathways. The olfactory route allows for faster drug delivery to the brain (taking approximately 0.33 h) compared to the trigeminal route (which takes about 1.7 h).^239^

However, when developing IN formulations, it is essential to consider the physiological characteristics of the nasal cavity. Challenges such as the limited volume for formulation application, mucociliary clearance, the mucus layer, and local enzyme activity can limit drug absorption via the IN route. Given these considerations, nanosystems have emerged as an effective strategy to enhance drug accumulation in the CNS.^237−240^ The design of nanocarriers is crucial in extending their residence time in the nasal cavity, enhancing their ability to penetrate the nasal mucosa, and ensuring targeted delivery to the brain. Therefore, it is essential to carefully select mucoadhesive polymers and surface-modified nanocarriers with specific ligands, such as peptides, to significantly improve the efficacy of this delivery route.^241^

In the context of GBM treatment, Hu *et al.* developed co-micelles containing cholesterol-conjugated AMO-21 (AMO-21c) mixed with T7c peptide. *In vivo* studies in rats showed that the AMO-21c/T7c co-micelles delivered AMO21 into the brain more efficiently than the control delivery systems. This effect was due to the T7 peptide, which increased binding to cells via TfRs on the cell surfaces. The tumor size was effectively reduced with IN administration of AMO-21c/T7c co-micelles compared to the other groups.^242^

In another study, Yang and co-workers constructed slit2 siRNA-loaded micelles by self-assembly of T7c in an aqueous solution. They demonstrated that T7c micelles could deliver siRNA to mouse brain tumor cells via IN administration. Additionally, T7c acted as both a GBM targeting peptide and an immune adjuvant, promoting dendritic cell proliferation and macrophage polarization.^243^ The same group used cholesterol-conjugated DP7 CPP enveloped with HA to create a siRNA (siVEGF or siPLK1) delivery carrier. *In vitro* studies demonstrated low cytotoxicity and high cell uptake efficiency. *In vivo* experiments demonstrated efficient siRNA delivery from the nose to the brain through the trigeminal pathway, which extended the lifespan of mice bearing glioma. Furthermore, intranasally administered drugs were exclusively detected in the brain, while intravenously administered drugs were found in other organs, demonstrating the reduced systemic side effects associated with IN delivery. The use of HA improved drug adhesion, prolonged retention time in the nasal cavity, prevented the cargo from entering the lungs, and increased mucosal permeability. Note that, while the DP7-C CPP was found to be effective for small RNA delivery and improved cellular uptake, it was the interaction between HA and CD44 that increased the accumulation of nanomicelles at the tumor site.^244^

Hu and colleagues designed a core–shell structured lipoplex loaded with c-Myc-targeting siRNA. R8 CPP was used to compact siRNA through electrostatic interaction, forming a core. A cationic liposome shell then encapsulated the core to prevent premature leakage of the cargo. Additionally, the lipoplex was modified with N9W-penetratin (89WP) CPP to enable penetration of the nasal mucosa. It was shown that the lipoplex could be preferentially internalized by glioma cells together with cell debris via active macropinocytosis. This uptake pathway prevented lysosomal entrapment of the lipoplex and increased its distribution in orthotopic glioma. Additionally, it released siRNA in the cytoplasm and significantly downregulated c-Myc mRNA and protein expression in glioma cells, thereby extending the lifespan of glioma-bearing mice.^245^

Okada and his group developed PEG–PCL micelles modified with TAT CPP as an IN delivery system of small molecules and gene therapy for GBM therapy.^246−248^ Nevertheless, enhancements were needed to ensure that drugs were selectively targeted to tumor sites upon reaching the brain while minimizing toxic side effects on healthy brain tissue. To increase the selectivity of this system to tumor cells, they modified CPT-loaded PEG–PCL-TAT micelles with Bombesin peptide (GQWAVGHLM), a GRPR peptide. Therefore, they could harness the overexpression of this receptor on the surface of GBM cells. *In vivo studies* demonstrated site directivity and superior therapeutic effects. Furthermore, IN administration of Bombesin peptide-functionalized micelles increased the median survival period of C6 glioma orthotopic bearing rats.^249^

#### 2.6.2 Local Treatment

As mentioned previously, the cornerstone of GBM treatment is maximal safe resection. This is attempted in all eligible patients to remove as much of the tumor as feasible while preserving neurologic function.^250^ The invasive nature of GBM makes complete surgical removal challenging, often resulting in residual infiltrating tumor cells. Consequently, tumor regrowth occurs in 95% of cases, with recurrences typically happening within 2–3 cm of the resection cavity.^9,251^ Therefore, delivering active agents locally within the tumor resection cavity has emerged as a viable option. This approach bypasses the BBB, allowing for therapeutic drug concentrations in the vicinity of residual tumor cells while minimizing or eliminating systemic side effects.^252^ Currently, the Gliadel wafer is the only FDA-approved local delivery implant for treating GBM. However, its clinical application has been limited due to issues with its stiffness and slow decomposition, as well as technical challenges during the implantation process.^253^

To overcome the problems of stiffness and slow degradation found in current implants, Gazaille *et al.* developed a polymer-free hydrogel composed of self-assembled gemcitabine-loaded LNC. Moreover, to improve the targeting of GBM cells and prevent off-target toxicities, LNC were functionalized with NFL peptide. *In vitro* studies showed that modified LNC had quicker internalization and increased cytotoxic effects. *In vivo* studies using a murine model of GBM resection demonstrated that functionalized gemcitabine-loaded LNC targeted nonresected GBM cells, significantly delaying or inhibiting the appearance of recurrences. The median survival with modified hydrogels was prolonged considerably compared to nonfunctionalized hydrogels (74 days *vs* 44 days, respectively).^254^

Kang and colleagues designed an injectable thermoresponsive hydrogel nanocomposite that incorporated DOX-loaded micelles and water-dispersible ferrimagnetic iron oxide nanocubes (wFIONs). To ensure drug targeting to GBM cells while reducing harm to healthy tissues, the backbone of the hydrogel was conjugated with T7 peptide. The micelles were designed to prevent premature drug release, target GBM cells, and release their drug payload in response to the acidic tumor microenvironment. Additionally, AMFs were utilized to induce mild HT and expedite the release and dispersion of micelles and cargo, enabling drug penetration to a depth of a few centimeters. *In vivo* studies conducted on an orthotopic mouse GBM resection model demonstrated significant tumor growth suppression, biocompatibility, and increased lifespan using this functionalized hydrogel.^255^

A hydrogel delivery system for the local release of a chemotherapeutic and immunoadjuvant combination through the resection cavity was developed in another study. The system comprised glutathione-responsive PTX-loaded NPs functionalized with the Pep-1 peptide and immunoadjuvant-loaded PLGA NPs. The NPs were rapidly gelled at the site of GBM resection and released continuously for 2 weeks. *In vitro* and *in vivo* studies demonstrated that this hydrogel could alter the tumor-suppressive microenvironment by activating NK cells and T cells while directly targeting residual GBM cells. The endocytosis of NPs through GBM cells was significantly enhanced by modification with the Pep-1 peptide. The modified NPs demonstrated higher cytotoxicity than the unmodified ones. *In vivo* experiments on rats that underwent tumor resection revealed that this system significantly extended their median survival time. Approximately 37.5% of the rats survived beyond 5 months after treatment.^256^

Wang *et al.* designed a 3D-printed hydrogel-liposome nanoplatform coloaded with TMZ and erastin and functionalized with cRGD peptide. *In vivo* results from a modified intracranial tumor resection model showed that the nanoplatform remained effective for over 14 days postapplication. Moreover, the functionalized nanosystem significantly extended the median survival time compared to that of the control groups. The study showed that the nanoplatform increased the sensitivity of glioblastoma cells to TMZ, resulting in antitumor efficacy while reducing the required dosage of TMZ and minimizing adverse side effects.^257^

## 3 The Gatekeeper in Distress: The Blood–Brain Barrier Disease Disruption

The BBB is a crucial interface between the systemic circulation and the brain parenchyma. It meticulously controls the passage of molecules into the CNS. However, the integrity of this barrier is subject to dynamic changes, which are particularly pronounced in various brain disorders.^258,259^ Alzheimer’s disease is characterized by the accumulation of β-amyloid plaques, which can induce inflammatory responses and oxidative stress. This can ultimately compromise the integrity of cerebral ECs. Similarly, neuroinflammation in Parkinson’s disease can disrupt tight junctions within cerebral ECs, leading to increased BBB permeability. Additionally, ischemic stroke and traumatic brain injury can cause acute disruption of BBB function, which may persist for several weeks after onset, further increasing its permeability.^260−262^ In the context of GBM, the expansion of the tumor within the brain parenchyma imposes spatial constraints, reducing the available free space and consequently disrupting the dynamics of blood flow. This phenomenon alters the geometry of cerebral vessels, increasing their tortuosity and thereby impeding the effective passage of nanocarriers across the BBB/BTB.^263^ In addition, GBM-associated neuroinflammation exacerbates BBB/BTB compromise by downregulating the expression of tight junction proteins in GBM-associated ECs, thereby increasing BBB/BTB permeability, as mentioned above. Notably, GBM has a distinct BBB/BTB profile characterized by the involvement of tumor-promoting GSCs. These cells possess the ability to differentiate into abnormal vascular pericytes, leading to aberrant astrocytic endfeet and proliferative responses. As a result, the expression of tumor growth-promoting factors is stimulated. In addition, angiogenic factors released by GBM cells serve a dual purpose: they not only support tumor expansion but also play a critical role in modulating BTB permeability.^264,265^

Delivery of therapeutics to the cerebral parenchyma has been shown to be effective using peptide-functionalized NPs, as discussed in the preceding sections. However, careful consideration of targeting peptide density and affinity is imperative to mitigate the risk of adherence to endothelial surfaces, which may result from high avidity interactions and consequently impede efficient BBB/BTB crossing. Therefore, optimizing the affinity and density of targeting molecules is critical to facilitate effective BBB/BTB penetration.^264^ Furthermore, the design of these nanosystem strategies must carefully consider parameters such as particle size and charge, as well as the temporal dynamics of BTB permeability, to ensure the exploitation of this transient window to access brain tissue. Understanding the disease-specific changes in BBB/BTB properties will be paramount in the design of NPs for efficient brain delivery.^263,264^

## 4 Challenges

**Figure 6:**
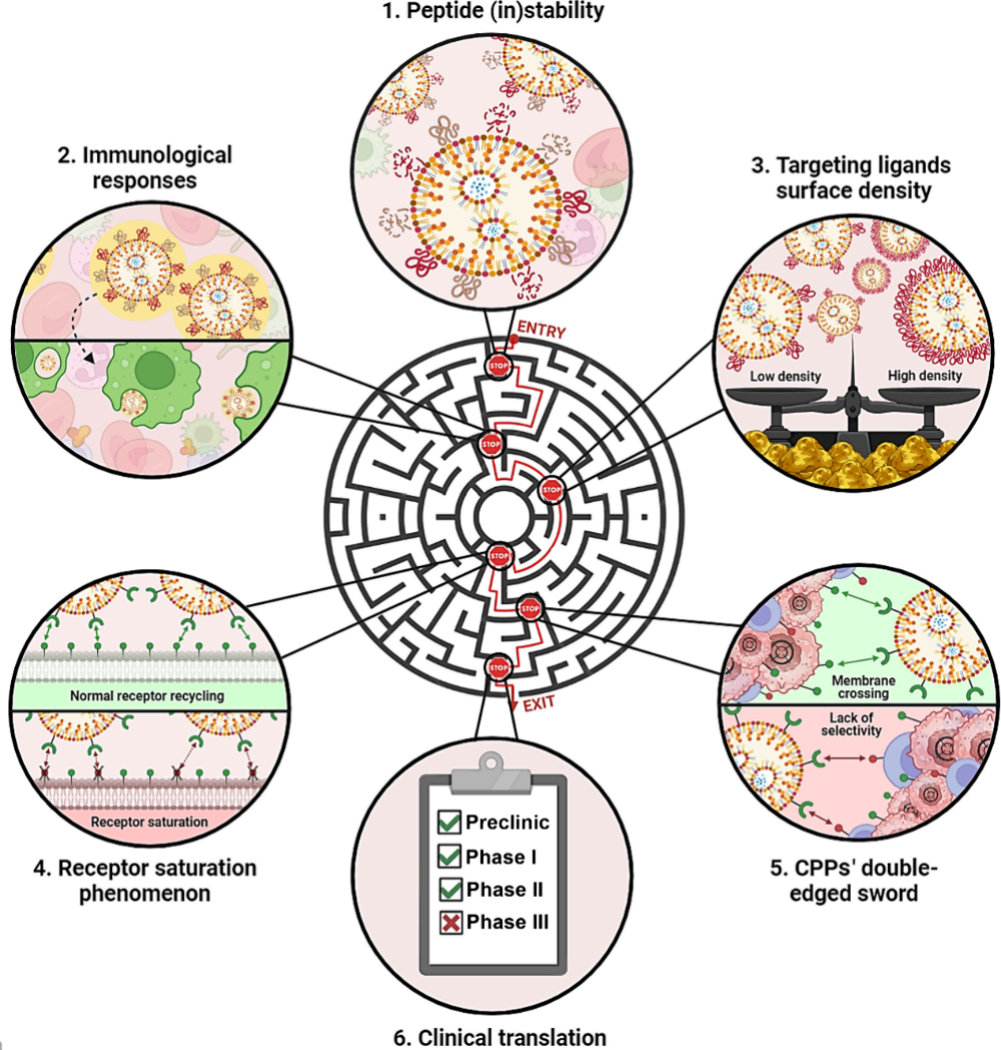
Challenges in designing peptide-decorated nanocarriers: navigating the maze. This figure highlights key challenges in developing peptide-decorated nanocarriers for targeted drug delivery. It includes peptide instability, immunogenicity, targeting ligand surface density, receptor saturation phenomenon, limitations in the specificity of CPPs, and considerations for clinical translation. Addressing these hurdles is crucial to advancing the effectiveness and safety of nanocarrier-based therapies in clinical applications. Abbreviations: CPP: Cell-penetrating peptide. (Created with BioRender.com).

The complex landscape of peptide-functionalized NPs in the context of GBM therapy unfolds a tapestry of challenges, covering stability and safety aspects and issues related to the development and up-scaling stages toward clinical application. These are explored in the sections that follow. (Figure 6)

### 4.1 (In)stability

Peptide-decorated nanocarriers represent a promising avenue for targeted drug delivery, leveraging the specificity of peptides for precise therapeutic interventions. However, the peptide stability *in vivo* is a critical and limiting step to their effectiveness.^266^ The inherent linear simplicity of the peptide structure, composed of L-amino acid residues, plays a pivotal role in its unique vulnerability to various forms of degradation. Deamidation, hydrolysis, and enzymatic degradation, mainly through proteolysis, are critical challenges that peptides face in biomedical applications. These processes can lead to significant structural alterations, especially when exposed to specific temperature and pH conditions. Consequently, such transformations can potentially compromise the efficacy and precision of nanocarriers engineered for targeted drug delivery. Addressing these issues through innovative peptide design and formulation strategies is imperative to harness the full potential of peptide-based nanomedicine.^26,267,268^

### 4.2 Immunological Responses

Once in the human body, peptide-decorated nanocarriers face challenges due to interactions with serum proteins, antibodies, and macrophages, which can lead to aggregation and degradation, directly affecting their therapeutic potential.^269^ Opsonins, including immunoglobulin G (IgG), complement factors, and fibrinogen, can trigger immune recognition, eliminating nanocarriers by the immune system.^270^ The intricate relationship between peptide properties and the immunocompatibility of peptide-decorated nanocarriers remains a frontier yet to be fully explored.^271−273^ Peptide molecular weight, structure, and origin are crucial factors that determine the similarity to host peptides, which in turn affects tolerance. It may influence the composition and properties of the protein corona formed around nanocarriers, introducing additional complexity.^274^ These coronas, derived from the adsorption of serum proteins, have profound implications ranging from increased visibility to macrophages and consequent accelerated clearance to reduced receptor recognition and, thus, reduced targeting capacity of nanocarriers.^270,275^ Their highly unpredictable nature requires a deeper understanding to anticipate and mitigate potential side effects, modulating their composition and stability. While the protein corona can be protective by shielding ligands from degradation, it poses potential challenges.^276^ Besides, forming several specific protein coronas on the surface of nanocarriers could guide them to target different organs.^277^ Structural changes and even denaturation of nanocarrier surfaces can occur in some cases, affecting their overall performance.^278^ Beyond surface modifications, components of the protein corona can initiate downstream processes, including activation of the complement system.^279^ These cascading events have far-reaching implications for nanocarrier functionality, affecting pharmacokinetics, immunogenicity, toxicity, and targeting efficiency.^280,281^

### 4.3 Receptor Saturation Phenomenon

The challenge of receptor saturation in the context of peptide-decorated nanomedicines targeting the brain is a critical obstacle to achieving optimal therapeutic outcomes. This phenomenon occurs when the available binding sites on target receptors become saturated with ligands, reducing the ability of nanocarriers to bind their intended targets effectively.^282^ Decorating nanocarriers with multiple ligands is a strategic approach to address this challenge and minimize receptor overload.^27^ It is also important to prioritize receptors or transporters less susceptible to saturation and undergo rapid recycling at the cell surface, ensuring sustained and efficient interaction with biological interfaces and optimizing the potential for therapeutic success.^117^ For example, while exogenous Tf may compete with native Tf for the same receptor site, resulting in a competitive inhibition effect, the T7 peptide presents a viable alternative to circumvent receptor saturation. This is because it has been demonstrated to possess equivalent targeting capabilities without competing with endogenous Tf.^51^ Following the same reasoning, Pep-1 also attaches to IL-13Rα2 at a different site from the native ligand and could be an excellent alternative to IL-13 peptide.^283^ Furthermore, the multireceptor binding properties of the ApoE peptide may reduce the load on their respective receptors (LDLR; LRP-1 and LRP-2), allowing time for them to undergo recycling, which may serve as a promising strategy to avoid saturation.^40,42^

### 4.4 Targeting Ligand Surface Density

Determining the ideal density of ligands is a complex task, especially when incorporating multiple ligands. This complexity arises because the optimal density varies depending on the specific types of ligands and receptors involved.^284,285^ Finding the right balance is critical: too many ligands can lead to nanocarrier aggregation, off-target interactions, or hindered binding due to physical obstacles, while too few can inhibit the uptake and internalization of nanocarriers by cells. Fine-tuning the density of ligands is essential to optimize the therapeutic potential of multiligand functionalized nanocarriers.^286,287^

### 4.5 Double-Edged Sword: The Case of CPPs

The incorporation of CPPs into nanocarriers introduces a double-edged sword in drug delivery. While CPPs facilitate the crossing of cellular barriers, their efficacy is membrane-dependent (cell type and specific membrane composition) and lacks the necessary selectivity for tumor cells. This nonspecific interaction raises concerns about unintended toxicity in nontargeted cells, undermining the precision of drug delivery. Careful consideration of membrane types and judicious selection of CPPs are essential to realize their potential while minimizing off-target effects.^27,101,288^

The clinical potential of CPPs for drug delivery may also be hampered by the rapid clearance through the reticuloendothelial system due to the cationic nature of these peptides. This limitation significantly impacts the efficacy of CPPs, as it can result in low bioavailability and poor uptake into target cells.^288−290^

### 4.6 From Bench to Bedside

The small size and increased surface area of drug nanocarriers make them well-suited for crossing biological barriers and reaching their intended targets. However, these properties might also be responsible for inducing toxicity, hindering the clinical translation of nanomedicines into practice, and limiting their market introduction.^291^ It is also of utmost importance to ensure that each NP component is nontoxic, biodegradable, or excreted from the body over time. Both Food and Drug Administration (FDA) and European Medicines Agency (EMA) guidelines emphasize the crucial management of peptide-related impurities and degradants arising from synthesis or degradation processes.^292,293^ These impurities pose safety risks, potentially eliciting immunogenic responses and carrying implications for drug safety and efficacy. Regulatory standards assign thorough reporting, identification, and qualification of impurities, guided by clinical and toxicological data for informed decision-making.^294,295^

Significant strides have been made in peptide-decorated nanocarrier research. However, the lack of standardized methods for their production and characterization remains a critical bottleneck in their clinical translation. Establishing reproducible and reliable protocols is essential to ensure the consistency of nanocarrier formulations required for regulatory approval and widespread clinical adoption. The standardization of production and characterization methods is a prerequisite for advancing the field and realizing the full therapeutic potential of these nanocarriers.^263,296^

The challenge of replicating natural tumors in laboratory models, particularly in the context of the human brain, poses a significant barrier to predicting the efficacy and safety of peptide-decorated nanocarriers in humans. Current knowledge predominantly relies on *in vitro* and *in vivo* models that often need to mimic the complexities of the human brain more accurately. For example, animal GBM cell lines fail to fully replicate the morphology and behavior of their human counterparts.^297,298^ Ensuring the reliability and reproducibility of data generated from *in vitro* and *in vivo* is imperative. Improved models that faithfully mimic the complexities of the human brain are essential for obtaining meaningful insights into the performance, safety, and efficacy of these nanocarriers.^275^ Organ-on-chip systems, organoids, and patient-derived xenograft models represent cutting-edge approaches that offer enhanced predictive capabilities, bringing us closer to translating preclinical findings into successful clinical outcomes.^3,299−301^ Scale-up, a pivotal aspect of clinical translation, introduces additional challenges in the context of peptide-decorated nanocarriers.^297^ Meeting regulatory standards is essential in nanomedicine development. Due to the complexity of this field, existing manufacturing processes might need adjustments, with a focus on creating robust and scalable production methods. This is important to ensure the safety and consistency of nanocarriers on a larger scale.^302^

Numerous clinical trials (CTs) exploring different approaches to treating GBM are showing promise for potential therapies.^303−306^ However, when it comes to strategies for functionalizing NPs with peptides, there are very few examples available. One promising advanced treatment option is 2B3–101, glutathione PEGylated liposomes that completed Phase I/IIa clinical trials for brain tumor therapy (NCT01386580). This formulation, loaded with DOX and featuring glutathione decoration, exhibited a 5-fold increase in brain delivery compared to conventional PEGylated liposomal DOX. The trial yielded promising results, demonstrating preliminary antitumor activity and a favorable safety profile in 28 patients. These findings suggest that the therapy may be effective and safe for treating brain tumors.^307−309^

Although the therapeutic potential demonstrated by peptide-decorated nanosystems, none have been approved for clinical use to date. In contrast, peptide-drug conjugates (PDCs) have led to the approval of two therapeutic agents currently available on the market, including Lutathera and Melflufen. Furthermore, other PDCs are currently being investigated and developed, including different stages of clinical trials.^310^ Furthermore, several Phase I CTs of GRN1005 (or ANG1005), a chemical entity combining ANG2 and PTX designed to penetrate the BBB via LRP1-mediated transport, have provided insight into its safety and biocompatibility, with predominant side effects attributed to PTX alone. Additionally, it has shown the ability to reach therapeutic concentrations in tumor tissue and to exert potent antitumor effects in recurrent glioma.^311^ The success of this peptide-based strategy could serve as a guiding principle for future research, development, and approval of peptide-decorated nanocarriers aimed at treating GBM.

## 5 Advanced Strategies for Surface Modification

### 5.1 Augmenting Therapeutic Viability through Peptide Stabilization

**Figure 7:**
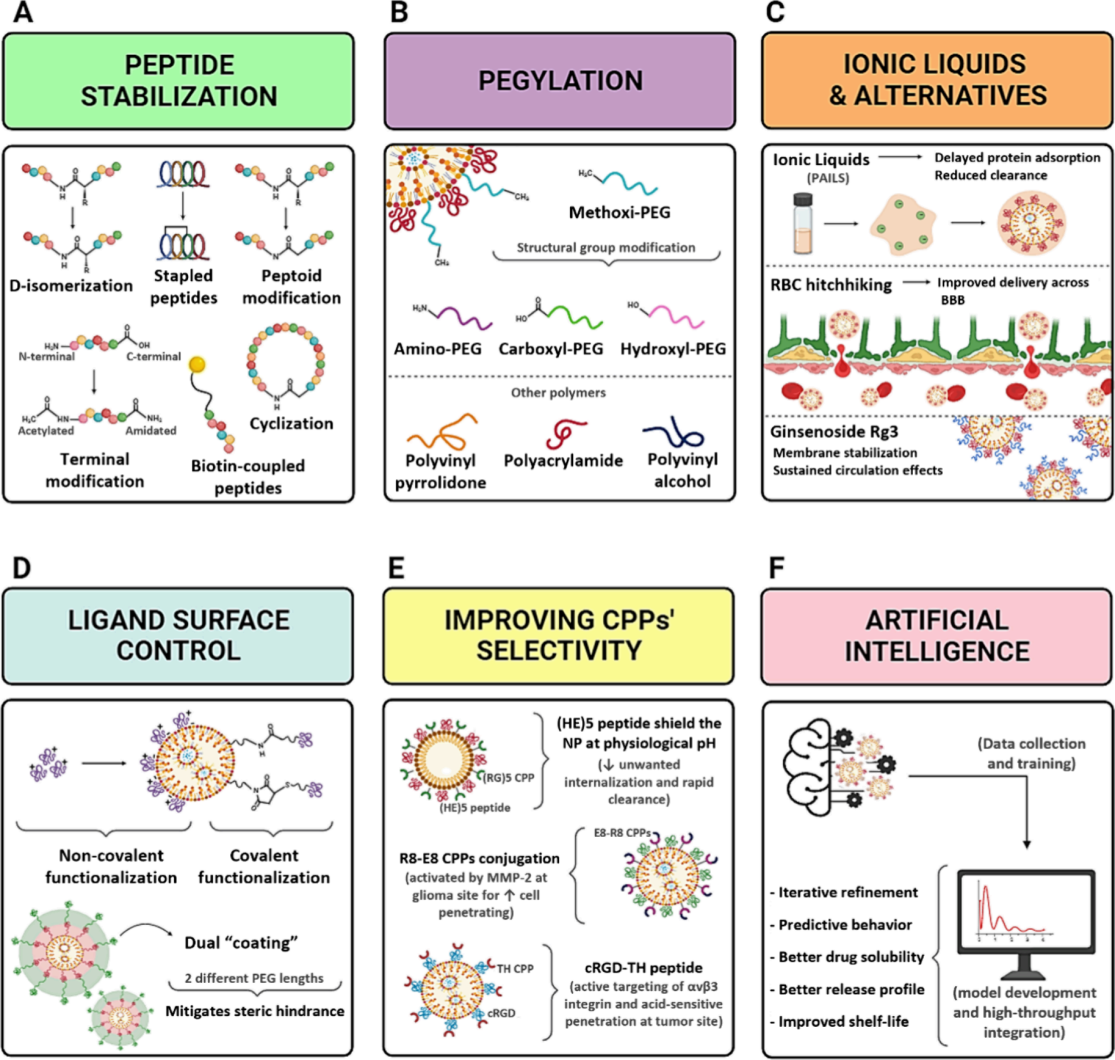
Strategies for overcoming peptide-decorated nanocarrier challenges. This figure illustrates different strategies to address challenges associated with peptide-decorated nanocarriers. Highlighted approaches include the integration of artificial intelligence and machine learning for optimized nanocarrier design, peptide stabilization through d-isomerization, stapled peptides, peptoid backbone modifications, terminal modifications, use of biotin-coupled peptides, and cyclization, modification of functional groups associated with PEG and the utilization of alternative polymers, the use of ionic liquids, control of ligand density and hindrance through dual-coating or covalent and noncovalent interactions and improvement of CPPs selectivity. Abbreviations: CPP: Cell-penetrating peptide; PAILs: Protein-avoidant ionic liquids; PEG: Polyethylene glycol. (Created with BioRender.com).

Among several approaches to enhance peptide stability and improve the targeting precision of peptide-decorated nanocarriers, cyclization is emerging as a key technique to convert linear peptides into closed-loop structures.^312^ (Figure 7A) The most used cyclic peptides in treating GBM are the cRGD peptides, as outlined in the preceding sections. These peptides exhibit enhanced stability and resistance to degradation, in contrast to linear RGD peptides, which are more susceptible to rapid breakdown and chemical degradation.^313−315^

The stapling process can also be regarded as a form of macrocyclization in which a confined cyclic unit is created. (Figure 7A) Stapled peptides use hydrocarbon linkers to connect two side chains of amino acids on the same side of a helix, thereby enhancing the stabilization of α-helices and cell permeability.^316−318^ Fadzen *et al.* developed a perfluoroaryl-stapled CPP that exhibited enhanced serum stability and higher brain uptake when conjugated with platinum (Pt)(IV).^319^ In addition, the conjugation of Pt(IV) to this macrocyclic-stapled CPP showed a significant increase in median survival time in a GBM xenograft murine model compared to controls.^320^ Besides, the sRAP12 peptide, designed by Ruan and co-workers, effectively enhanced the degree of α-helix of RAP12, thus increasing proteolytic resistance, serum stability, and LRP1-targeting affinity compared to the RAP12 peptide.^45^ The same team also developed a sRGD peptide that showed improved targeting properties, as previously detailed.^83^

Enhancing peptide stability can also involve a classic approach known as terminal modification. (Figure 7A) Since exopeptidases target peptide bonds from either the N- or C-termini, strategies such as N-terminal acetyl capping and C-terminal amidation enhance resistance to exopeptidase degradation.^321^ Another alternative approach involves a fundamental modification of the peptide backbone. Some researchers have explored using peptoid backbones instead of traditional peptide backbones, offering simplified synthesis processes, increased proteolytic stability, and improved performance in cellular uptake.^322^

The stereochemical arrangement of amino acids plays a crucial role in substrate recognition and binding to proteases. Consequently, substituting l-isomers with d-isomers is emerging as a potent strategy to effectively impede enzymatic degradation and thereby prolong the half-life of peptides in physiological fluids.^312^ (Figure 7A) For example, ^D^CDX is a D-peptide that is highly stable in lysosomes or serum and enhances BBB transcytosis. Although it raised some immunogenicity concerns compared to the original ^L^CDX peptide, the ^D^CDX peptide holds excellent promise for brain-targeted drug delivery and GBM treatment.^88^ For instance, D-AE, an enantiomer of the L-AE peptide, was designed by replacing L-amino acids with D-amino acids without changing the sequence. Results showed that D-AE retained the multifunctional tumor-targeting efficacy of the parent peptide L-AE while exhibiting enhanced metabolic stability.^93^ Wei and colleagues also created a retro-inverso peptide of ANG2 (^L^Angiopep), known as ^D^Angiopep, which showed resistance to proteolysis in fresh rat serum. In contrast, over 85% of ^L^Angiopep degraded within 2 h.^323^ RI-VAP (^D^P^D^A^D^V^D^R^D^T^D^N^D^S), the retro-inverso peptide of the L-VAP peptide, has also shown higher stability relative to its origin, along with excellent binding affinity to GRP78.^100^

Moreover, modifying certain CPPs, such as NFL and TAT, with biotin has proven to be a successful strategy in improving the stability of these peptides, influenced by chemical configurations and steric arrangements of peptides.^324,325^ (Figure 7A) All these innovative strategies represent a paradigm shift in addressing the challenges of peptide stability and targeting precision.

### 5.2 PEGylation

The performance of nanocarriers in drug delivery is intricately tied to their interactions with the immune system, specifically the formation of the protein corona and the subsequent recognition by opsonins.^271,326,327^ Conversely, deopsonins, such as serum albumin and lipoproteins, play a crucial role in blocking immune recognition, prolonging blood circulation time, and enhancing drug delivery efficiency.^270^

Functional coatings are promising for improving drug targeting and administration by modulating immune recognition. Polymers with stealth properties, such as PEG, have been extensively used to limit opsonin adsorption, decreasing nanocarrier clearance.^328^ PEG also improves formulation stability during storage by preventing nanocarrier aggregation.^329^ However, PEG is a nondegradable polymer and may lead to immunogenic responses against PEGylated NPs by the increasing prevalence of anti-PEG antibodies present in the bloodstream.^330−333^ The widespread administration of Pfizer and Moderna’s COVID-19 vaccines, delivered in PEGylated lipid NPs, has raised concerns about the accelerated development of these anti-PEG antibodies in the vaccinated population.^331,334^ Therefore, exploring alternatives is necessary to provide greater stability and mitigate immunogenicity concerns, some of which have shown success. PEG can be structurally modified by replacing methoxy-PEG with other functional groups such as amino (−NH_2_), carboxyl (−COOH), and hydroxyl (−OH) or by using other polymers such as polyvinylpyrrolidone, poly(vinyl alcohol) or polyacrylamide.^332^ (Figure 7B) However, none of the alternatives are immunologically inert.^334^ Additionally, some ongoing studies aim to develop alternative polymers with longer circulation times to address the trade-off between achieving stealth properties and maintaining targeting capabilities.^335,336^

Regarding multiligand strategies, manipulating the outer arms of polymers like PEG allows the construction of nanocarrier surfaces with variable lengths of each ligand. This innovative approach facilitates fine-tuning nanocarrier properties, optimizing their performance for specific therapeutic applications.^124,337^ With this in mind, Martins *et al.* used a dual coating strategy with two different PEG lengths, creating two distinct coatings with specific functions. The long PEG paired with the ANG2 peptide forms the first/external coating, targeting the BBB. Upon reaching the acidic pH of endosomes, the PEGylated ANG2 peptide cleaves from the NP, facilitating endosomal escape. As a result, l-Histidine coupled with the short-length PEG is exposed to target its receptors in GBM cells.^118^

### 5.3 Ionic Liquids and Other Alternatives

Ionic liquids (ILs) are a class of nonmolecular compounds composed solely of ions. What sets them apart is their unique property of melting at temperatures below 100 °C. ILs have gained significant attention and popularity due to their ability to serve as versatile solvents, catalysts, and electrolytes in various processes. Their low melting points make them practical and accessible for a wide range of reactions, while their ionic nature allows them to dissolve a diverse array of compounds.^338^ Using ILs represents a promising strategy for functionalizing nanocarriers, offering an alternative to traditional approaches such as PEGylation. (Figure 7C) ILs can potentially reduce protein adsorption and opsonization, thereby improving the biocompatibility of nanocarriers.^339,340^ Hamadani *et al.* specifically used ILs with low protein solubility, known as protein-avoiding ionic liquids (PAILs), to coat NPs and developed an innovative approach characterized by delayed adsorption of proteins on the surface of NPs. This reduction of protein adsorption appears to be a promising alternative to PEG, resulting in better performance than conventional PEGylated NPs.^341^ Another strategy focuses on “RBC hitchhiking” to take advantage of the natural properties of erythrocytes and allow them to travel “stealthily” to the brain. (Figure 7C) ILs can “hitchhike” on RBCs. This innovative approach could significantly improve the delivery efficiency of nanocarriers across the BBB, with approximately 15–20% of ILs-coated NPs reaching the BBB compared to less than 1% for ligand-decorated NPs.^341,342^ ILs may also assist in crossing the BBB. For instance, taking advantage of the overexpression of choline transporters in the BBB, choline-2-hexanoic acid IL may facilitate the transport of nanocarriers across the barrier.^342^ Therefore, the hypothesis is that combining the immune evasion of ILs with the targeting precision of peptides on nanocarriers represents an ideal synergistic strategy: ILs would reduce immune recognition and enhance BBB accumulation. In contrast, peptides would contribute to specific targeting of tumor cells, collectively enhancing the bioavailability and therapeutic efficacy of nanocarriers.

Xia *et al.* innovatively designed liposomes by incorporating ginsenoside Rg3 instead of PEG and cholesterol. Ginsenoside Rg3 demonstrated membrane stabilization capabilities comparable to cholesterol and provided a sustained circulation effect similar to PEG. (Figure 7C) This approach holds great promise for prolonging the circulation time of the liposomes in the blood, thereby mitigating potential immune responses and the phenomenon of accelerated blood clearance.^343^

Finding the right balance between biocompatibility and selectivity potential is crucial in the design of nanocarrier coatings. Doing so with membranes derived from macrophages, leukocytes, erythrocytes, and even tumor cells has emerged as a promising strategy for effective tumor targeting and immune system evasion.^197,302,344−346^ In addition, using endogenous proteins such as albumin, ferritin, and ApoB as carriers is also an efficient approach, underscored by its nonimmunogenic and nontoxic properties.^199−201^

Furthermore, developing nanocarrier systems that respond to specific stimuli (enzymes, pH, light) presents an intriguing avenue. Such systems can dynamically adjust their ligand presentation during transport to the target site. For instance, in the context of GBM treatment, nanocarriers could selectively expose ligands for GBM cell selection while in transit, ensuring optimal targeting efficiency upon reaching the tumor site.^347,348^ Exploring innovative approaches to enhance BBB targeting, some studies propose using ligands that cleave at the acidic pH of endosomal vesicles within the barrier.^258,349^ The cleavage mechanism prevents strong binding to receptors, reducing the risk of rapid degradation of transported nanosystems in endothelial cells. Instead, it promotes an intermediate binding force, forming syndapin-2 tubular structures and accelerating the transfer of nanosystems across the BBB.^350^ Given the considerable heterogeneity of GBM in terms of acidic regions within the tumor microenvironment, caution is advised in the design of such strategies. This heterogeneity varies between patients and individual gliomas, emphasizing the critical need for precise design strategies.^351,352^

Concerning protein corona, the functionalization of NPs with stealth polymers has proven effective in reducing protein adsorption to their surface, although complete prevention remains a challenge.^353^ Current research is actively exploring strategies to modify NP surfaces by incorporating antibodies, ligands, and functional proteins such as albumin, apolipoproteins, or clusters of differentiation 47 (CD47). These efforts aim to hamper the formation of the protein corona and address its associated limitations, optimizing the biological response to NPs and improving their efficacy and safety.^276^

### 5.4 Control of Ligand Surface: Density and Hindrance

The successful development of nanocarriers for targeted drug delivery hinges on meticulously managing the quantity and variety of ligands on their surfaces.

Strategies for functionalizing nanocarriers with peptides tailored for tumor targeting include both covalent and noncovalent methods. (Figure 7D) Covalent attachment of peptides to NPs is a leading approach due to its high stability and precise control over targeting site selectivity.^354−356^ Conde *et al.* proposed a strategy to functionalize polyester aminic NPs with iNGRt peptides to target the NRP-1 receptor in the spectrum of noncovalent approaches. In this case, the goal was the treatment of breast cancer, but NRP-1 receptors are overexpressed in several cancers, including GBM. The peptides were linked to a poly(glutamic acid) chain or a palmitoyl chain and associated with NPs through electrostatic or hydrophobic bonds, respectively. The latter strategy showed the most promising results. NPs decorated with palmitoylated peptides showed a strong association with the ligands, ensuring their correct exposure and orientation on the NP surface. This is important for effective targeting and binding to specific receptors, relieving some potential steric hindrance.^357^ Other studies confirmed the improved stability, enhanced cellular uptake, and better maintenance of monodispersity and coating in the NPs functionalized with palmitoylated peptides.^358^

In addition, a notable consideration in the design of nanocarriers is the potential challenge of steric hindrance resulting from a high density of ligands on their surface. This phenomenon can hinder or reduce the interaction between ligands and their corresponding receptors.^359^ To address this issue, the strategy of dual coatings with different PEG lengths developed by Martins *et al.* provides an interesting example. (Figure 7D) Besides the advantages of targeting and specificity, this innovative design also mitigates steric hindrance. Upon cleavage between the first coating layer (PEGylated ANG2 peptide) and the NP, a structural rearrangement occurs, exposing the inner coating layer (PEGylated l-Histidine). This dynamic rearrangement reduces the likelihood of steric hindrance and enhances the interaction between the ligands and their respective receptors for effective targeted drug delivery.^118^

### 5.5 Improving CPPs Selectivity

The broad cellular internalization capability of CPPs raises concerns about potential cellular toxicity due to the uptake of therapeutics by normal tissues.^322^ A promising strategy involves the design of activatable CPPs (ACPPs) to address this. Due to their molecular structure, the penetrating activity of ACPPs is initially suppressed to prevent nonspecific cellular uptake. The ACPP structure includes a polycationic active domain with cell-penetrating ability, a cleavable connecting arm, and a polyanionic shielding domain. The polycationic part interacts electrostatically with the polyanionic part, temporarily inhibiting cell internalization. However, it can be conditionally activated in response to chemical or biological stimuli, such as enzymes, pH, light, or exogenous substances that cleave this interaction. With this innovative strategy, other possibilities have emerged to refine the selectivity and efficacy of nanocarrier-based drug delivery systems.^360^ The previously described strategy used by Tian *et al.* showed that the polyanionic masking (HE)_5_ peptide introduced into the micelle conjugated with the CPP (RG)_5_ served as a shield at physiological pH. (Figure 7E) The results showed that it was able to effectively minimize the cationic charges of CPP (RG)_5_, avoiding unwanted internalization and rapid clearance.^119^ Also, Gu *et al.* designed an ALWMP in which the cationic LMWP was initially masked by the polyanionic peptide E10 via an MMP-sensitive linker sequence. In the tumor environment, the linker was selectively cleaved, thereby exposing LMWP to target GBM cells.^120^ Besides, in the approach above designed by Gao and co-workers, the anionic E8 was used to hinder the cationic property of R8 through an MMP-2 sensitive linker, which further exposed R8 CPP in the tumor site, circumventing their poor selectivity and improving their penetrating properties.^125^

Tandem combinations also showed the potential to overcome the lack of selectivity of CPPs. For example, Liu and co-workers designed a tandem peptide conjugating c(RGD) in the front position and R8 CPP in the back end. (Figure 7E) This strategy takes advantage of the specific targeting of integrin receptors by c(RGD) and the high penetrating capacity of R8, and the results showed that this design could overcome the bottleneck of the nonspecific penetrating capacity of CPP, increasing GBM site accumulation while reducing toxicity in other organs.^126^ Moreover, the TR peptide - a combination of c(RGDfk) and TH CPP - clearly demonstrated a responsive tandem peptide. (Figure 7E) Upon internalization and in response to the acid microenvironment, the inactive TR peptide showed a proton sponge effect, which enabled the CPP properties of TH CPP and allowed a deep penetration into the GBM cells.^131^ In another study, to prevent unintended penetration during blood circulation, the TAT CPP was linked to a short PEG2000 chain, which was then covered by a longer PEG6000 chain. This structure enabled the ANG2 peptide to be fully exposed on the surface, while the TAT peptide was concealed within the longer PEG layers. Following the binding of ANG2 to its receptor, the TAT peptide was brought close to the cell membrane, subsequently initiating its entry into the cell.^124^

### 5.6 Artificial Intelligence Combined with High-Throughput Methods

Achieving the desired performance of nanocarriers requires a highly delicate balance, and manufacturing conditions such as flow rates and ligand conjugation ratios play a critical role in determining their physicochemical properties. Size, uniformity, surface charge, and ligand density all influence therapeutic efficacy and require precise control during formulation.^284,361^

Several techniques have been employed to screen and evaluate peptides that bind functional NPs, such as cell surface display, peptide array methods, and phage display. However, these methods can be resource-intensive and time-consuming, which may impede the rapid development of effective drug delivery systems.^362−364^ The rise of artificial intelligence (AI), particularly machine learning (ML), is introducing a paradigm shift in nanocarrier development.^365,366^ (Figure 7F) ML-based models, trained on existing data, can predict formulation behavior, leading to improved drug solubility, consistent release profiles, and extended shelf life. The value of ML lies in its ability to refine formulations iteratively by incorporating information on both successful and unsuccessful outcomes, thereby increasing the efficiency of the development process.^367,368^ For instance, by integrating ML with high-throughput techniques, researchers can rapidly identify promising formulations and materials. This accelerates the selection of NPs with optimal properties for various applications, reducing the development timeline and improving the accuracy and success rate of nanocarrier formulations.^369,370^

Since peptide discovery remains a significant bottleneck in developing modified nanocarriers, identifying the essential factors that influence the binding between peptides and nanocarriers can be challenging. A proof-of-concept study employing ML was carried out to accelerate the analysis of NP-binding peptides. Using unsupervised learning methods like k-means, hierarchical, and Louvain clustering, more than 1720 gold-binding peptides were clustered to identify those with stronger binding affinity to gold NPs. Peptide clustering identified essential features of peptides, particularly isoelectric points, in modulating their binding with gold NPs. The data set was divided into two parts: 80% for the training set and 20% for the testing set to facilitate the training of different supervised learning models. Therefore, the performance of the supervised models was assessed in terms of metrics, demonstrating that supervised learning models such as notably logistic regression, decision tree, random forest, k-nearest neighbors, naïve Bayes, support vector machine, and neural network could effectively predict peptide binding properties. The approach developed in this study has the potential for future applications in targeting diseases such as GBM by enabling precise peptide selection and design.^371^

Furthermore, AI techniques have the potential to extract data-driven insights from omics data and analyze patient-specific information. This can assist in designing peptides that are customized to individual patients’ tumor profiles.^372,373^ The concept of digital twins can be applied to translate AI analyses for use in personalized medicine. This enables the selection of the appropriate functionalized drug for each patient based on their characteristics.^374^

The complex and dynamic nature of the protein corona makes it a challenge to describe and predict its composition accurately. AI, especially ML algorithms, can examine large data sets and help identify specific proteins within the corona, elucidate their roles, and detect patterns in protein-NP interactions to predict their potential impact on nanocarrier behavior in biological systems.^375,376^

## 6 Drilling down the Findings

In this section, a comprehensive summary of peptide functionalization strategies employed in the treatment of GBM is presented. An attempt to categorize and analyze a diverse range of NP approaches is done, detailing the specific peptides used, their corresponding receptors, surface targeting locations/overexpression sites (BBB, GBM cells, or GSCs), associated physical stimuli (such as HT, PTT, magnetic delivery, US, RT), nanocatalytic medicine or biomimetic alternatives, providing insights into the current clinical status of these promising therapeutic strategies. (Table 1)

**Table 1:**
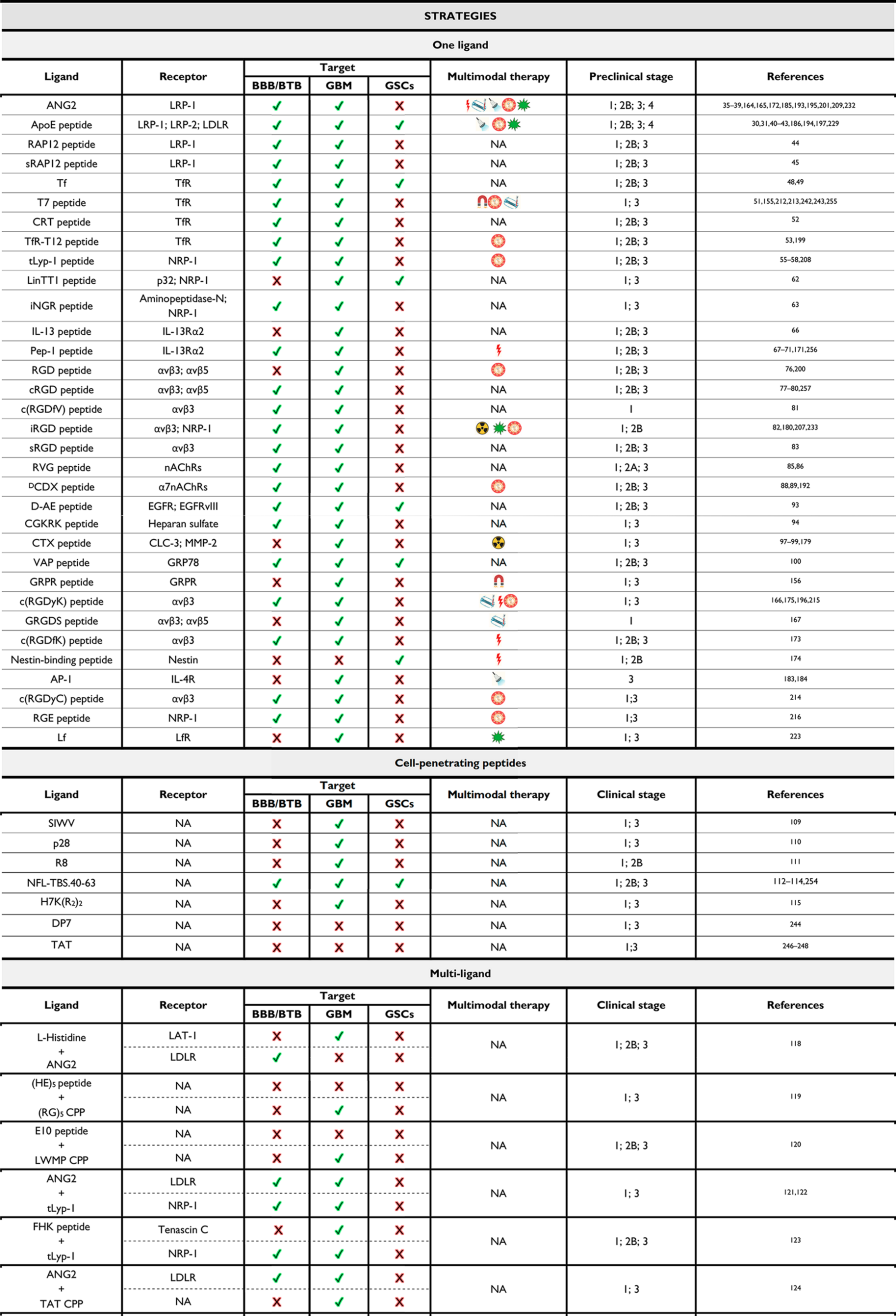

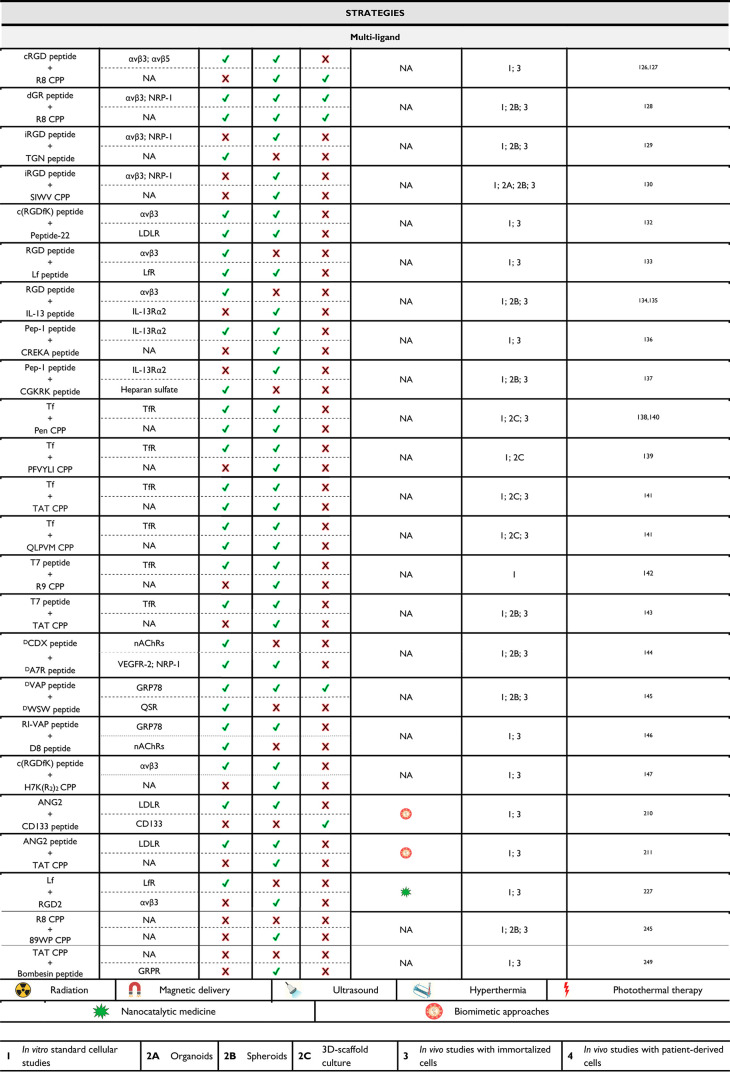
Summary of the Various Therapeutic Approaches Based on Peptide-Functionalized NPs Mentioned^a^

a Abbreviations: ANG2: Angiopep-2; AP-1: Atherosclerotic plaque-specific peptide-; ApoE: Apolipoprotein E; BBB: Blood-brain barrier; BTB: Blood-tumor barrier; CLC-3: Chloride channel-3; CPP: Cell-penetrating peptide; CTX: Chlorotoxin; EGFR: Epidermal growth factor receptor; EGFRvIII: Epidermal growth factor receptor variant III; GSCs: Glioma stem cells; GRPR: Gastrin-releasing peptide receptor; IL-13: Interleukin 13; L-13Rα2: Interleukin 13 receptor alpha 2; IL-4R: Interleukin 4 receptor; LAT-1: L-type amino acid transporter 1; LDLR: low-density lipoprotein receptor; Lf: Lactoferrin; LfR: Lactoferrin receptor; LRP-1: LDLR-related protein-1; LRP-2: Megalin; LWMP: Low-weight molecular protein; MMP-2: Matrix metalloproteinase 2; NA: Not applicable; nAChRs: Nicotinic acetylcholine receptors; NRP-1: Neuropilin-1; QSR: Quorum sensing receptor; RGD2: RGD peptide dimer; Tf: Transferrin; TfR: Transferrin receptor; VEGFR-2: Vascular endothelial growth factor receptor 2.

## 7 Conclusion

In summary, addressing the complex challenge of GBM requires innovative, multifaceted approaches to effectively combat its inherent aggressiveness and therapeutic resistance. This review highlights the significant potential of peptide-functionalized nanocarriers in the treatment of GBM. Given its status as the most common malignant tumor of the CNS and its notoriously rapid progression, peptide-functionalized NPs offer substantial promise in the quest for effective treatment options. The exploration of multitargeting ligands has shown great promise in GBM treatment, particularly when compared to single-targeting approaches. These ligands simultaneously use different peptides to engage a range of overexpressed receptors. These advanced strategies enhance the precision of drug delivery, facilitate BBB penetration, and enable targeting specific molecular pathways within the complex microenvironment of GBM. The synergistic use of peptides with external stimuli offers a robust approach that combines precise targeting with the diverse functionalities of external agents. In addition, biomimetic strategies, characterized by reduced immunogenicity and sometimes intrinsic targeting capabilities, also showed encouraging results in GBM therapy. Moreover, the emerging field of nanocatalytic medicine, which exploits the catalytic properties of NPs to generate cytotoxic agents while enhancing targeting efficiency through peptide decoration, represents a promising direction away from conventional chemotherapy. There has also been considerable research into alternative routes of parenteral administration. Nose-to-brain delivery and local delivery within the resected tumor cavity have been shown to bypass the BBB and have emerged as promising routes to be explored in the context of peptide decoration for GBM. Future research should focus on further elucidating the overexpressed receptors in GBM and on developing peptides or combination therapies that specifically target these receptors for improved treatment efficacy. Despite the challenges associated with peptide degradation and formulation with nanomedicines, advances in pharmaceutical technology and bioengineering offer viable solutions to these obstacles. Innovative methodologies, including the application of AI and ML, will play a pivotal role in advancing the field and translating these therapeutic strategies into the clinic. In the ongoing battle against GBM, the strategic integration of peptide-functionalized NPs into treatment paradigms represents a significant leap forward that could offer patients a more effective treatment option. Their specificity and ability to address the unique challenges of GBM position these NPs as essential tools for improving patient outcomes and providing a glimmer of hope in the daunting fight against this disease.

## Vocabulary

Glioblastoma: the most prevalent and lethal of the brain cancers, classified as a grade IV glioma.
Blood–brain barrier: a protective barrier that regulates the flow of substances between the bloodstream and the brain.
Nanoparticles: particles that are either unbound or aggregated, with at least one external dimension falling within the size range of 1–100 nm for more than 50% of the particles.
Peptides: short chains of amino acids (typically 2–50) linked by peptide bonds.
Biomimetic approaches: materials or systems designed for various applications, such as drug delivery, by mimicking biological processes or structures for better biocompatibility and efficacy.
Nanocatalytic medicine: a treatment modality that uses nanomaterials as catalysts to generate reactive oxygen species.
